# Gut feelings: associations of emotions and emotion regulation with the gut microbiome in women

**DOI:** 10.1017/S0033291723000612

**Published:** 2023-11

**Authors:** Shanlin Ke, Anne-Josee Guimond, Shelley S. Tworoger, Tianyi Huang, Andrew T. Chan, Yang-Yu Liu, Laura D. Kubzansky

**Affiliations:** 1 Channing Division of Network Medicine, Department of Medicine, Brigham and Women's Hospital and Harvard Medical School, Boston, MA 02115, USA; 2 Department of Social and Behavioral Sciences, Harvard T.H. Chan School of Public Health, Boston, MA 02115, USA; 3 Lee Kum Sheung Center for Health and Happiness, Harvard T.H. Chan School of Public Health, 677 Huntington Avenue, Boston, MA 02115, USA; 4 Department of Cancer Epidemiology, Moffitt Cancer Center and Research Institute, Tampa, FL 33612, USA; 5 Department of Epidemiology, Harvard T.H. Chan School of Public Health, Boston, MA 02115, USA; 6 Clinical and Translational Epidemiology Unit, Massachusetts General Hospital and Harvard Medical School, Boston, MA 02114, USA; 7 Center for Artificial Intelligence and Modeling, The Carl R. Woese Institute for Genomic Biology, University of Illinois at Urbana-Champaign, Champaign, IL 61801, USA

**Keywords:** Emotion regulation, emotions, gut–brain axis, gut microbiome

## Abstract

**Background:**

Accumulating evidence suggests that positive and negative emotions, as well as emotion regulation, play key roles in human health and disease. Recent work has shown the gut microbiome is important in modulating mental and physical health through the gut–brain axis. Yet, its association with emotions and emotion regulation are understudied. Here we examined whether positive and negative emotions, as well as two emotion regulation strategies (i.e. cognitive reappraisal and suppression), were associated with the gut microbiome composition and functional pathways in healthy women.

**Methods:**

Participants were from the Mind-Body Study (*N* = 206, mean age = 61), a sub-study of the Nurses' Health Study II cohort. In 2013, participants completed measures of emotion-related factors. Two pairs of stool samples were collected, 6 months apart, 3 months after emotion-related factors measures were completed. Analyses examined associations of emotion-related factors with gut microbial diversity, overall microbiome structure, and specific species/pathways and adjusted for relevant covariates.

**Results:**

Alpha diversity was negatively associated with suppression. In multivariate analysis, positive emotions were inversely associated with the relative abundance of *Firmicutes bacterium CAG 94* and *Ruminococcaceae bacterium D16*, while negative emotions were directly correlated with the relative abundance of these same species. At the metabolic pathway level, negative emotions were inversely related to the biosynthesis of pantothenate, coenzyme A, and adenosine.

**Conclusions:**

These findings offer human evidence supporting linkages of emotions and related regulatory processes with the gut microbiome and highlight the importance of incorporating the gut microbiome in our understanding of emotion-related factors and their associations with physical health.

## Introduction

Mounting evidence supports the role of emotion-related factors in disease etiology and health promotion (Kubzansky et al., 2018; Levine et al., 2021). Both negative (e.g. depression, anxiety) and positive (e.g. happiness, pleasure) manifestations of emotion have been linked with maintaining physical health as well as the risk of developing disease, including cardiovascular disease (CVD) (Boehm & Kubzansky, 2012; Levine et al., 2021), obesity (Mannan, Mamun, Doi, & Clavarino, 2016), and overall mortality (Trudel-Fitzgerald et al., 2019). Additional work suggests emotion regulation – that is, the strategies by which individuals shape the nature of emotions they experience as well as when and how they experience these emotions – may also impact health and help explain observed associations of distinct positive and negative emotions with a range of health outcomes (Trudel-Fitzgerald, Guimond, & Kubzansky, in press).

The human body and particularly the gastrointestinal (GI) tract is inhabited by hundreds of trillions of microbes, collectively known as the human microbiome. Rather than simple passengers in or on our bodies, commensal microbes play key roles in physical health and diseases (Clemente, Ursell, Parfrey, & Knight, 2012). A disrupted gut microbiome is associated with various health conditions including cardiometabolic diseases (Brial, Le Lay, Dumas, & Gauguier, 2018). Accumulating evidence suggests bidirectional communications between gut microbiota and the brain, i.e. the gut–brain axis, which plays a key role in mental and physical health (Morais, Schreiber, & Mazmanian, 2020). The gut–brain axis consists of bidirectional communication among the central nervous system (including brain regions involved in emotion processing and emotion regulation, notably the amygdala, the hippocampus, and the prefrontal cortex), the autonomic nervous system, and the enteric nervous system (Carbia et al., 2021; Morais et al., 2020). Thus, the gut–brain axis links emotional and cognitive areas in the central nervous system with the gut; this connection allows bidirectional effects whereby the brain can drive changes in the gut environment and alter the microbial composition; and the gut microbiota can in turn influence emotional processes. Prior work has found multiple psychiatric disorders characterized by frequent and intense negative emotions (e.g. depression, anxiety) are often accompanied by functional GI disorders (Muscatello, Bruno, Scimeca, Pandolfo, & Zoccali, 2014) and perturbations of the gut microbiome (Nikolova et al., 2021; Yang et al., 2020), suggesting associations between emotion-related factors and the gut microbiome are likely. A recent meta-analysis found that patients with a range of psychiatric disorders shared similar patterns of gut microbiome perturbations, including depletion of certain anti-inflammatory bacteria and enrichment of pro-inflammatory bacteria (Nikolova et al., 2021). Taken together, these studies suggest certain gut microbial species or functions could be a key mechanistic pathway by which emotion-related factors contribute to physical health outcomes.

Existing research has mostly focused on populations of patients with discrete, identifiable psychiatric conditions. Yet, subclinical affective disturbances such as the tendency to experience negative emotions (e.g. sadness, fearfulness) are more common than psychiatric conditions, and are associated with worse physical health outcomes similar to full-blown psychiatric disorders (Cohen & Rodriguez, 1995; Muscatello et al., 2014; Nabi et al., 2008). Further, scholars have posited that both subclinical and clinical affective disturbances influence physical health through common biological pathways (Cohen & Rodriguez, 1995). Thus, gut microbiome perturbations might be linked with negative emotions even in non-clinical populations. Only one study to date has evaluated links between subclinical levels of negative affect and the gut microbiome. This small cross-sectional study evaluated associations of both negative and positive emotions with several microbial features within a Korean cohort (*n* = 83) using 16S rRNA gene sequencing. Results showed higher positive emotion but not negative emotion scores were related to *Prevotella* enterotype and higher alpha diversity (i.e. within-sample taxonomic diversity), while both higher positive emotion scores and lower negative emotion scores were linked with a genus from the family Lachnospiraceae (Lee et al., 2020). However, 16S rRNA gene sequencing typically yields general taxonomic profiling (e.g. family or genus), potentially missing associations with specific microbial species and pathways. Moreover, this study did not account for important confounders of the emotion–microbiome association such as health status and diet.

While previous research has established associations between the gut microbiome and brain structures involved in emotion regulation (Carbia et al., 2021), links between the gut microbiome and specific emotion regulation strategies are currently unexplored. Emotion regulation is a higher-order, transdiagnostic process that involves both up- and down-regulation of positive and negative emotions, and scholars have posited this process may explain how and why both positive and negative emotions appear to predict physical health outcomes (Trudel-Fitzgerald et al., 2019). Existing health research has mostly focused on two emotion regulation strategies: cognitive reappraisal (reinterpreting the meaning of an event to alter emotional responses before they occur) and suppression (inhibiting emotional behavior after emotions occur). Evidence generally suggests reappraisal is beneficial for physical health, while suppression is generally deemed more maladaptive for health (Trudel-Fitzgerald et al., 2019). Past work suggests links between emotion regulation strategies and gut function. Notably, suppression has been associated with exacerbated symptoms of irritable bowel syndrome such as delayed gut transit, abdominal pain, and increased postprandial colonic motility (Bennett et al., 2000; Evans, Bennett, Bak, Tennant, & Kellow, 1996). However, no work has yet examined if specific gut microbial features are associated with suppression and cognitive reappraisal. Identifying plausible mechanistic pathways linking emotion-related factors and health is necessary for establishing causality in emotion–health associations and understanding how interventions targeting emotional functioning can protect health.

In the current study, we examined associations of positive and negative emotions, as well as two emotion regulation strategies (i.e. cognitive reappraisal and suppression) with gut microbiota compositions and functional pathways. To do this, we used an agnostic approach to evaluate emotion-related factors with respect to gut microbiome features using data from an ongoing cohort of older women. Based on previous work suggesting positive emotions and cognitive reappraisal are generally health-protective, and negative emotions and suppression are associated with poorer health outcomes (Levine et al., 2021; Trudel-Fitzgerald et al., 2019), we expected different associations between these two sets of processes with features of the gut microbiome. However, given the paucity of previous research, we did not have *a priori* hypotheses regarding the nature and magnitude of such associations.

## Methods

### Study population

**Fig. 1.**
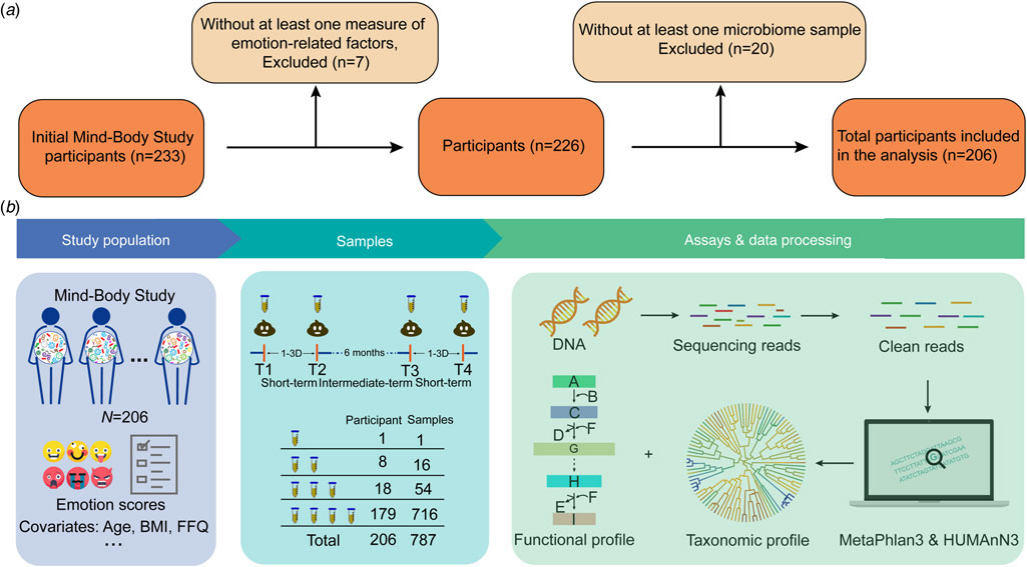
Conceptual framework of the study. This analysis was designed to evaluate the associations of emotion-related factors (i.e. positive and negative emotions, as well as emotion regulation strategies), with the gut microbiome. (a) The exclusion process for participants and the microbiome samples. (b) The analytic sample included 206 women from the MBS, nested within the NHSII cohort. Each participant provided up to four stool samples; one pair of stool samples was collected 24–72 h apart about 3 months after the questionnaire was administered followed by a second pair about 6 months later. Phenotypic data were collected through a mailed questionnaire assessment, including individual characteristics, emotion-related factors, health conditions, and health behaviors including habitual dietary intake. DNA was extracted from all fecal samples. The taxonomic and functional profiling were performed using MetaPhlAn3 and HUMAnN3, respectively. D, day; BMI, body mass index; FFQ, food-frequency questionnaire.

Data are from a sub-study (MBS: Mind-Body Study, *n* = 233) within the Nurses' Health Study II (NHSII) cohort (Huang et al., 2019), an ongoing prospective cohort study of 116,  429 US female registered nurses. At enrollment in 1989, participants completed a questionnaire reporting on their demographics, lifestyle factors, and medical history. Biennial follow-up questionnaires have been mailed to all participants to update exposure information and disease diagnoses. Return of the completed questionnaires implied consent to use the data in ongoing research. At the study baseline in 2013, MBS participants (age: 49–67 years) signed a written informed consent form and completed a comprehensive online psychosocial assessment including measures of emotion-related factors, i.e. positive and negative emotions as well as emotion regulation (Fig. 1). The same measures were completed again 1 year later but because fewer women were missing data on the emotion-related variables at the baseline assessment, we chose to use only baseline measures in our main analyses to preserve statistical power. However, to verify whether emotion-related factors scores remained stable across the two measurements available, we calculated the intraclass correlation coefficients (ICCs) for each factor and provided them in the Results section. Approximately 3 months after the initial psychosocial survey, participants were invited to provide up to four stool samples. Obtaining repeated samples over these relatively short intervals was designed to help reduce measurement error rather than to look at the change in microbiome composition over time. Women were eligible for inclusion in the current study if they completed at least one relevant emotion-related measure and provided at least one valid stool sample (*N* = 206). Among the 206 eligible participants, 179 (18, 8, and 1) participants provided 4 (3, 2, and 1) stool samples, respectively. This yielded 787 stool samples in total. The study protocol was approved by the institutional review boards of the Brigham and Women's Hospital and Harvard T.H. Chan School of Public Health.

## Emotion-related factors

We were interested in assessing links between the gut microbiome and positive and negative emotions across the spectrum, rather than examining only psychopathology. To do so, we used items from the 10-item Center for Epidemiological Studies Depression (CES-D-10) Scale (Irwin, Artin, & Oxman, 1999) and the Kessler Psychological Distress Scale, 6-item version (K-6) (Kessler et al., 2002) to derive separate measures of positive and negative emotions. Following prior use of these instruments (Yiengprugsawan, Kelly, & Tawatsupa, 2014), we combined sets of items to create a continuous measure across levels of either positive or negative emotions rather than using any kind of cut-point to create a measure of probable psychopathology.

### Positive emotions

Self-reported positive emotions were derived from two positively worded items (During the past month… ‘I was happy’, ‘I felt hopeful about the future’) from the CES-D-10. Items were scored on a 4-point Likert scale. Items were averaged with higher scores indicating higher positive emotions levels. A positive emotions score was assigned if participants completed both items (*n* = 202). Internal consistency was acceptable (*α* = 0.75). Past work supports a two-factor structure for the CESD-10, with these two positively worded items loading on a positive affect factor (Bradley, Bagnell, & Brannen, 2010; Irwin et al., 1999). These items have been used in several studies examining the role of positive emotions in health outcomes (Boehm et al., 2020; Wilson et al., 2017). The 30-day timeframe allowed our measure to be sensitive to both situational and trait factors (Mroczek & Kolarz, 1998). We standardized the positive emotions score for analyses (mean = 0, s.d. = 1).

### Negative emotions

We created an overall measure of negative emotions by pulling items from self-reported measures administered in MBS. Negative emotions were derived from combining data on seven items: four CESD-10 items (During the past month… ‘I was bothered by things that usually don't bother me’, ‘I felt depressed’, ‘I felt fearful’, ‘I was lonely’), and three non-redundant K-6 items (During the past month, about how often did you feel…‘nervous’, ‘hopeless’, ‘restless’, or ‘fidgety’). The CESD-10 items were scored on a 4-point Likert scale with response options ranging from ‘Rarely or none of the time’ to ‘All of the time’. The K-6 items were rated on a 5-point Likert with response options ranging from ‘None of the time’ to ‘All of the time’. To harmonize the K-6 items with the CESD-10 items, we combined two response options (i.e. ‘Some of time’ and ‘A little of the time’) to create a 4-point Likert scale resulting in a similar set of response options. Items were averaged with higher scores indicating higher levels of negative emotions. A negative emotion score was created if participants completed five items (*n* = 206). Internal consistency of the seven items was acceptable (*α* = 0.77). Negative emotions scores were standardized for analyses (*M* = 0, s.d. = 1).

### Emotion regulation strategies

Participants completed the Emotion Regulation Questionnaire, a validated 10-item scale that measures cognitive reappraisal (six items) and emotional suppression (four items) (Gross & John, 2003). Items are scored on a 7-point Likert scale ranging from 1 (‘strongly disagree’) to 7 (‘strongly agree’). A total score is obtained for each subscale by averaging the items of the subscale, with higher scores reflecting higher use of each emotion regulation strategy. A cognitive reappraisal score was created if participants completed at least five items (*n* = 204). For suppression, a score was created if participants completed at least three items (*n* = 204). Internal consistency in this sample was good for the cognitive reappraisal subscale (*α* = 0.87) and acceptable for the suppression subscale (*α* = 0.76). Cognitive reappraisal and suppression scores were standardized for analyses (mean = 0, s.d. = 1).

### Sample collection, DNA extraction, and metagenome sequencing

From 2013 to 2014, each participant provided up to two pairs of stool samples (Fig. 1). Each pair of stool samples was collected from two bowel movements 24 to 72 h apart. The second set of two samples was collected ~6 months later. Collection kits were mailed to participants with detailed instructions and returned to our biorepository via overnight courier within 24 h of collection. See Huang et al. (2019) for further details on sample collection.

DNA purification from stool aliquots was performed according to standard protocols used in the Human Microbiome Project (Human Microbiome Project, 2012; Integrative, 2019). Following previous work in MBS, construction and sequencing of sample libraries were conducted at the Broad Institute (Wang et al., 2021b). Specifically, metagenome libraries were constructed using the Illumina TruSeq or Nextera method with ~180 nt inserts and sequenced on one of the Illumina HiSeq platforms (2500 or 4000) targeting a minimum of ~2 Gnt/sample with 100 nt paired-end reads.

### Microbiome taxonomic and functional potential profiling

For the raw metagenomics sequencing data, low-quality reads were discarded, and reads belonging to the human genome were removed by mapping the data to the human reference genome with KneadData (Mehta et al., 2018). Microbial taxonomic profiling was performed using MetaPhlAn3 (Beghini et al., 2021). We then performed functional profiling for metagenomes by applying HUMAnN3 (Beghini et al., 2021), which maps DNA reads to a customized database of functionally annotated pan-genomes.

### Covariates

Covariates included factors that can be related to both emotion-related factors and the gut microbiome; all were self-reported on the MBS questionnaire unless otherwise stated. Sociodemographics included age (continuous; based on the date of birth queried at NHSII baseline in 1989), race/ethnicity (White, racially underrepresented individuals; queried in 2005 on the NHSII biennial questionnaire), marital status (married/cohabitating, never married/divorced/widow; queried in 2013 on the NHSII biennial questionnaire), husband's education (unmarried participant, husband: less than high school, high school graduate, college graduate, and graduate school; queried in 1999 on the NHSII biennial questionnaire), and census-tract income (continuous, geocoded in 2001). Health-related factors (yes, no) included the history of diabetes and hypertension (queried on every NHSII biennial questionnaire), hormone therapy use, and antidepressant use. Body mass index (BMI) in kg/m^2^ was derived from height (queried in 1989) and weight (Rimm et al., 1990). Alcohol consumption in g/day was derived from dietary information collected in 2013 using a validated semi-quantitative food-frequency questionnaire (FFQ) (Willett et al., 1985). Weekly physical activity was determined according to the number of min/week women reported spending in moderate to vigorous activity, e.g. running (<150 min/week, >150 min/week; queried on the 2013 NHSII biennial questionnaire) (Wolf et al., 1994). Using dietary information obtained from the FFQ, we computed the Alternative Healthy Eating Index 2010 score (AHEI-2010, a score that measures adherence to a diet pattern based on foods and nutrients most predictive of chronic disease risk) (Chiuve et al., 2012). See the online Supplementary material for additional information.

### Statistical analysis

Descriptive analyses were conducted to assess the distribution of participants' characteristics as well as emotion-related factors mean levels, intercorrelations (using Spearman correlations), and ICCs. Microbial diversity measures were calculated at the species and metabolic pathway levels, using the ‘vegan’ R (v.2.5-7) package. We used the Richness, Evenness, Shannon, and Simpson diversity indices to determine the alpha diversity. We then calculated Spearman correlations between emotion-related factors and the alpha diversity. If participants provided multiple microbiome samples (*n* = 205), we computed the average alpha diversity across samples for each participant. For beta diversity for community composition and functional capacity over time, we used the Bray–Curtis (BC) dissimilarity and principal coordinates analysis (PCoA). Because the microbiome samples were collected at multiple time points, we assessed if the microbiome features changed meaningfully over time using a permutational multivariate analysis of variance (PERMANOVA) performed with the ‘adonis’ function in R's vegan package. See the online Supplementary material for additional information.

To evaluate associations of sociodemographic, health-related, and behavior-related covariates with the gut microbiome overall structure, we performed omnibus testing with PERMANOVA of BC dissimilarity (9999 permutations) to quantify the size and significance of the effect of each covariate in relation to the gut microbial composition and metabolic pathways. Only those covariates that were found to be significantly associated with the gut microbial composition and metabolic pathways at all four time points were kept in the multivariate linear mixed analyses described below.

In analyses testing our primary research questions, we used multivariate linear-mixed models in MaAsLin2 (microbiome multivariable associations with linear models) (Mallick et al., 2021) to evaluate associations of gut microbial species and pathways with emotion-related factors while adjusting for the covariates that showed significant associations with the gut microbial composition and metabolic pathways. These models included each participant's identifier as a random effect to account for within-individual correlation in microbiome metrics that could occur due to the study's repeated sampling design, as well as occasional missing observation of microbiome samples at some time points. Nominal *p* values across all associations were then adjusted for multiple comparisons using the Benjamini–Hochberg method with a target rate of 0.25 for *q* values (Wang et al., 2021a). Statistical analyses were performed with R (v.3.6.3) and SAS (v.9.4, 2013).

## Results

### Characteristics of study population

Women were on average 60.7 years old in 2013 (s.d. = 3.8; range = 49.4–66.8). Most participants were White (96%) and married/cohabitating (78%). Mean BMI was 26.4 kg/m^2^, 33% of women reported a history of hypertension, and 25% reported using antidepressant medication; see Table 1. Higher positive emotion scores were inversely correlated with lower negative emotion and suppression scores (*r* = −0.49, *p* < 0.0001 and −0.30, *p* < 0.0001, respectively), and positively correlated with higher cognitive reappraisal scores (*r* = 0.24, *p* = 0.001). Higher negative emotion scores were also correlated with higher suppression scores (*r* = 0.28, *p* < 0.0001); see online Supplementary Table S1. ICCs ranged from 0.65 (suppression) to 0.87 (positive and negative emotions), suggesting the emotion-related factors scores were reasonably stable over time.

**Table 1.** Characteristics of the study population at 2013 baseline (*N* = 206)

Age (years, mean ± s.d.)	60.7 ± 3.8
Race, %	
White	96
Black	3
Ethnicity, %	
Non-Hispanic	97
Hispanic	2
Husband's education level, %	
Less than high school	2
High school degree	15
College degree	43
Graduate degree	39
Census-tract income in thousands of $US, mean ± s.d.	67.7 ± 27.0
Married/cohabitating, %	78
Hypertension history, %	33
Diabetes history, %	5
BMI (kg/m^2^, mean ± s.d.)	26.4 ± 6.1
Currently using hormone replacement therapy, 0 = no, 1 = yes, %	24
Alcohol consumption (g/day, mean ± s.d.)	7.8 ± 10.8
⩾2.5 h/week (150 min) of moderate or vigorous activity, %	65
Current smoker, %	3
AHEI (mean ± s.d.)	52.7 (44.6)
Currently using antidepressant medication, %	25
Emotion-related factors (raw scores)	
Positive emotions (mean ± s.d.)	2.28 ± 0.61
Negative emotions (mean ± s.d.)	0.75 ± 0.37
Cognitive reappraisal (mean ± s.d.)	30.98 ± 5.81
Suppression (mean ± s.d.)	12.12 ± 4.61

*Note.* Values are means (s.d.) for continuous variables; percentages for categorical variables. Values of polytomous variables may not sum to 100% due to rounding.

BMI, body mass index; AHEI, alternate healthy eating index-2010.

### Emotion-related factors and microbiome diversity

**Fig. 2.**
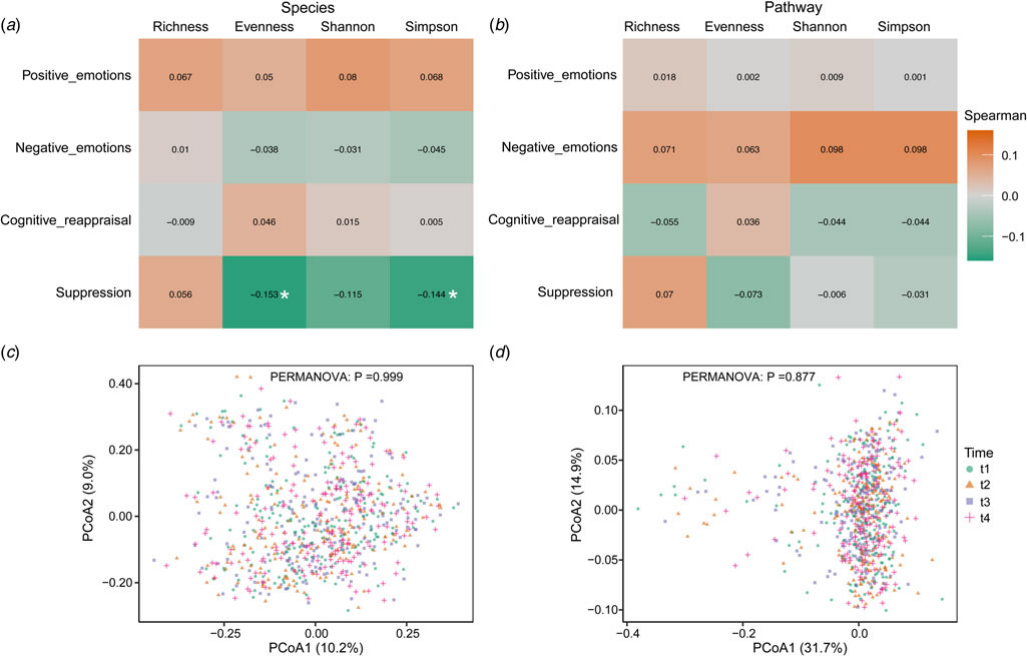
Association between emotion-related factors and gut microbial diversity. The heatmap displays the correlation between average alpha diversity and emotion-related factors at the species (a) and metabolic pathway (b) levels. Correlations were determined by Spearman correlations and asterisks denote statistically significant associations (*p* ⩽  0.05). Associations in this panel were conducted based on the average alpha diversity of the microbiome sample collected from 206 participants. PCoA of all microbiome samples over time at (c) the species and (d) the metabolic pathway levels based on BC dissimilarity. Analyses in panels (c–d) were conducted based on all 787 metagenomes collected from 206 participants. All PERMANOVA tests were performed with 9999 permutations based on BC dissimilarity.

We identified 467 microbial species and 479 metabolic pathways across all microbiome samples. Higher levels of suppression were significantly associated with lower Evenness and Simpson diversity at the species level (Fig. 2a). However, the alpha diversity was not significantly correlated with positive emotions, negative emotions, or cognitive reappraisal. We found no significant associations at the metabolic pathway level. When examining changes in microbiome features over time, we found no significant differences over time with respect to both species (Fig. 2c, *p* = 0.999) and metabolic pathways (Fig. 2d, *p* = 0.877). This is concordant with prior work with the same sampling strategy showing that the male adult gut microbiome remains relatively stable over time (Mehta et al., 2018).

### Associations between host factors, emotion-related factors, and overall gut microbiome structures

**Fig. 3.**
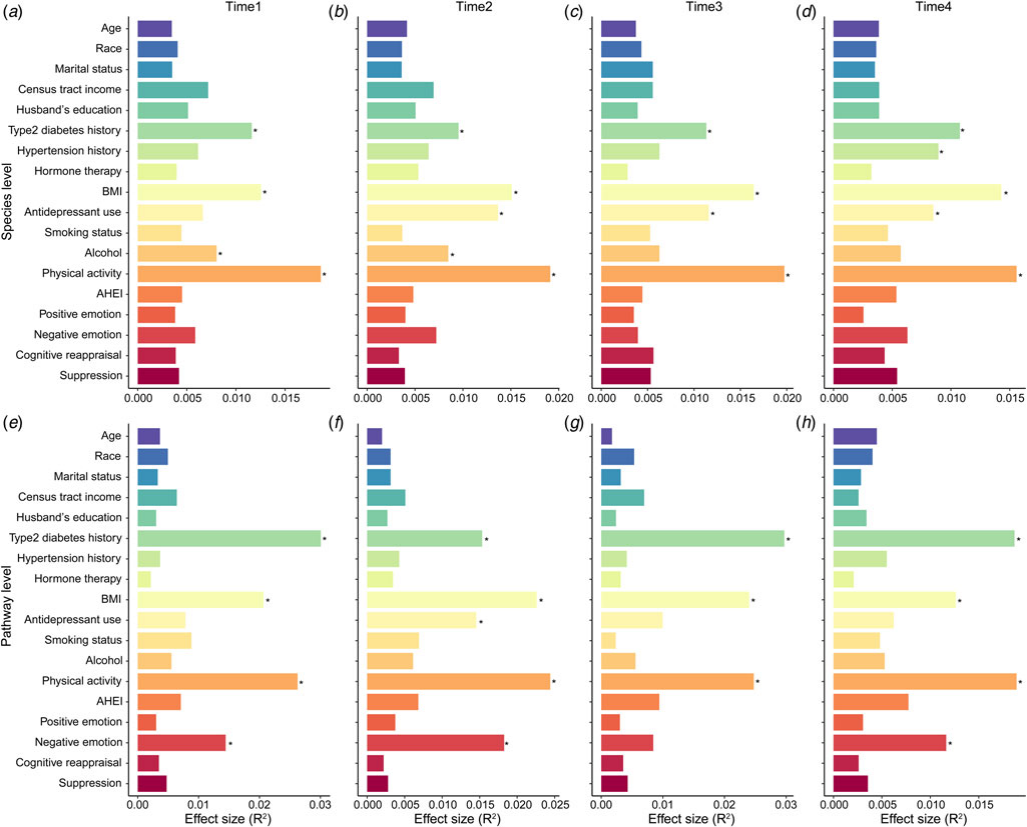
Gut microbiome-associated host factors. The amount of variance (*r*^2^) explained by each host factor in the taxonomic (at the species level, a–d) and functional (at the metabolic pathway level, e–h) profiles was determined by PERMANOVA analysis. All analyses were conducted based on all 787 metagenomes collected from four time points of 206 participants. The size of microbiome samples collected at first (a, e), second (b, f), third (c, g), and fourth (d, h) time points are 203, 197, 193, and 194. The asterisks denote significant associations (*p* ⩽ 0.05). AHEI, alternate healthy eating index. The color of each bar represents a different host factor.

Three host factors (i.e. physical activity, BMI, and type 2 diabetes history) were significantly associated with overall microbiome structure in both taxonomic and functional profiles at each time point (Fig. 3). Among all host factors considered, physical activity accounted for the largest proportion of variation in the species-level taxonomic profiles (Fig. 3a–d). It also explained the greatest amount of variance of the functional profiles at metabolic pathway level from the second (Fig. 3f) and fourth time points (Fig. 3h). We also found that negative emotion scores were significantly associated with overall microbiome structure at the metabolic pathway level at three time points (Fig. 3e, f, h). Positive emotion, cognitive reappraisal, and suppression scores were not significantly associated with overall microbiome configurations.

### Associations of emotion-related factors with species-level features and metabolic pathways of the gut microbiome

**Fig. 4.**
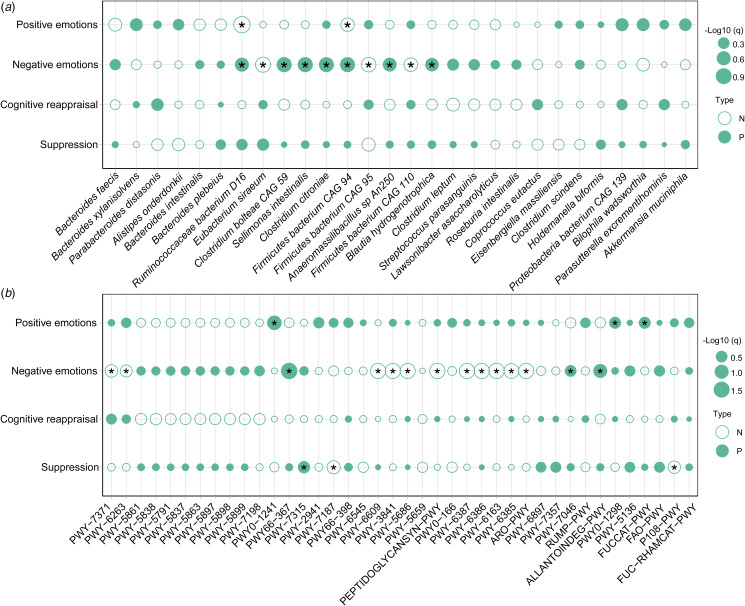
Associations of emotion-related factors with the human microbiome. Significant associations between positive and negative emotions and emotion regulation strategies and microbial species (a) and functional pathways (b) were identified using MaAsLin2. Top 10 features from each emotion-related factor are summarized (based on *q* value). Filled and hollow circles represent positive and negative associations, respectively. Only statistically significant associations with *q* value ⩽ 0.25 (Benjamini–Hochberg-adjusted *p* value) are labeled with a star. The size of each dot represents the −log 10 (*q* value). All analyses were conducted based on all 787 metagenomes collected from 206 participants. See online Supplementary Table S2 for coefficients and exact *p* and *q* values on these microbial species. See online Supplementary Table S3 for coefficients, exact *p* and *q* values and annotations of these metabolic pathways.

Top 10 species-level features from each emotion-related factor are summarized in Fig. 4a and online Supplementary Table S2. Positive emotions were significantly and inversely associated with *Firmicutes bacterium CAG 94* and *Ruminococcaceae bacterium D16*, while negative emotions were significantly related to higher abundance of these same species (*q* ⩽ 0.25). Moreover, we found some species shared consistent relationships with emotions and emotion regulation strategies in the expected directions and with similar magnitude of association, although not all *q* values reached statistical significance. For example, *Bacteroides xylanisolvens*, *F. bacterium CAG 9*5, and *Parabacteroides distasonis* were correlated with higher level of both positive emotions and cognitive reappraisal, as well as inversely correlated with both negative emotions and suppression. Further, positive emotions and cognitive reappraisal were associated with lower abundance of multiple species (i.e. *Anaeromassilibacillus* sp. *An250*, *Bacteroides faecis*, *Blautia hydrogenotrophica*, *Clostridium bolteae CAG 59*, *Clostridium leptum*, *F. bacterium CAG 94*, *R. bacterium D16*, *Sellimonas intestinalis*, and *Streptococcus parasanguinis*), while negative emotions and suppression were correlated with higher abundance of these species.

Top 10 metabolic pathways-level features from each emotion-related factor are summarized in Fig. 4b and online Supplementary Table S3. Positive emotions were significantly associated with higher abundance of three pathways (PWY0-1241: ADP-l-glycero-*β*-d-manno-heptose biosynthesis, PWY0-1298: superpathway of pyrimidine deoxyribonucleosides degradation, and FUCCAT-PWY: fucose degradation). Negative emotions were associated with a total of 63 metabolic pathways (Fig. 4b and online Supplementary Table S4, *q* ⩽ 0.25). For example, negative emotion scores were significantly inversely correlated with pathways related to coenzyme A (CoA) biosynthesis (e.g. CoA-PWY-1, PANTOSYN-PWY, CoA-PWY, and PWY-4242), adenosine biosynthesis (e.g. PWY-6609, PWY-7219, PWY-7229, and PWY-6126), and pyrimidine deoxyribonucleosides salvage (PWY-7199). Suppression scores were negatively associated with pyrimidine deoxyribonucleotides *de novo* biosynthesis II (PWY-7187) and a pathway related to the biosynthesis of propanoate (P108–PWY, Fig. 4b). We found no significant associations between cognitive reappraisal and any metabolic pathway.

## Discussion

In this discovery-based study, we examined associations between four emotion-related factors and the gut microbiome, leveraging a richly characterized cohort of 206 women. This study is the largest to date to evaluate associations of the gut microbiome with both positive and negative emotions and to assess the gut microbiome in relation to emotion regulation strategies. We found specific emotion-related factors were linked to microbiome diversity, as well as with certain species and metabolic pathways. These findings collectively offer early evidence suggesting emotions and emotion regulation strategies are related to the gut microbiome.

A prior study identified the significant association of microbial diversity (i.e. Shannon diversity) with positive (but not negative) emotions among participants in the *Prevotella*-enterotype group. Moreover, both positive and negative emotions were associated with a novel genus (*PAC001043_g*) from the family Lachnospiraceae (Lee et al., 2020). Among the emotion-related factors included in our study, only suppression was significantly associated with alpha diversity. Notably, higher levels of suppression were associated with lower values on the Simpson diversity index and less Evenness in the gut microbial community at the species level. The reason why only suppression was associated with diversity indexes is unclear. However, this finding is consistent with previous work showing that suppression and reappraisal influence health outcomes and biological processes differently, with suppression having stronger effects on the physiological outcomes examined thus far (e.g. increased sympathetic activation) than reappraisal (Gross, 1998). However, further work is needed to replicate our findings and understand the mechanisms underlying the differential associations of emotion-related factors with alpha diversity.

We also considered microbiome associations at the species and metabolic pathway levels. These analyses consistently demonstrated specific microbial associations of positive emotions and cognitive reappraisal in one direction and opposite associations with negative emotions and suppression. For example, lower levels of positive emotions and higher levels of negative emotions were associated with increased *F. bacterium CAG 94* and *R. bacterium D16*. Interestingly, a multi-study, integrative analysis on 4347 human stool metagenomes from 34 published studies including healthy and unhealthy individuals (e.g. having CVD, colorectal cancer, obesity) found *R. bacterium D16* was less prevalent in healthy (*vs.* unhealthy) individuals (Gupta et al., 2020). Similarly, we observed that higher levels of positive emotions and cognitive reappraisal were inversely correlated with multiple other species (e.g. *Anaeromassilibacillus* sp. *An250*, *B. faecis*, *B. hydrogenotrophica*, *C. bolteae CAG 59*, *C. leptum*, *S. intestinalis*, and *S. parasanguinis*), while higher levels of negative emotions and suppression were associated with increased abundance of these same species. Some of these species were previously reported as being associated with disease related to both mental health and inflammatory disorders, conditions that have also been linked with emotional distress and dysregulation (Guimond, Kubzansky, & Lee, 2021). For example, *C. bolteae* has been found elevated in neuromyelitis optica spectrum disorders (Pandit et al., 2021), and *S. intestinalis* was found enriched in the gut microbiota of patients with schizophrenia (Thirion et al., 2022). *S. parasanguinis* is a dominant isolate of dental plaque and an opportunistic pathogen associated with subacute endocarditis (Chen et al., 2020). Taken together, these findings suggest favorable emotional functioning, characterized by higher levels of positive emotions and lower levels of negative emotions, as well as better emotion regulation (i.e. greater use of reappraisal, and lower use of suppression), are associated with distinct compositional profiles of the gut microbiome at the species level. More research is needed to better understand the role of specific species in health, particularly for outcomes such as CVD that have been related to emotions and emotion regulation.

When examining metabolic pathways, higher levels of negative emotions were associated with lower abundance of metabolic pathways in the biosynthesis of pantothenate and CoA. Pantothenate (i.e. vitamin B5) is the key precursor for the biosynthesis of CoA, a universal and essential cofactor involved in many metabolic reactions, including phospholipid synthesis, biosynthesis and degradation of fatty acids, and the tricarboxylic acid cycle (Leonardi & Jackowski, 2007). Our results are consistent with prior work that found moderate intake of pantothenic acid was related to lower odds of experiencing anxiety symptoms (Mahdavifar, Hosseinzadeh, Salehi-Abargouei, Mirzaei, & Vafa, 2021). In addition, we found multiple metabolic pathways related to adenosine biosynthesis were significantly and inversely correlated with negative emotions. This is concordant with the fact that adenosine, one of the most ubiquitous and conserved neuromodulators in the central nervous system (Sperlagh & Sylvester Vizi, 2011), may have beneficial impacts on depressive and anxious symptomatology (Gomes et al., 2021). However, how disruption of those metabolic pathways may interact with emotion-related factors warrants further investigation.

Our study has several limitations. First, this study was conducted among 206 adult women who are health professionals and mostly White; moreover, a relatively large proportion of our analytic sample was using antidepressant medication. Therefore, the generalizability of our findings needs to be further validated by external studies based on larger and more diverse populations of women and men of different ages. Second, while bidirectionality in the association of emotion-related factors with the microbiome is likely, we cannot test for causality nor directionality in these relationships within this cross-sectional study. Our findings do identify rigorously assessed associations considering within-person correlation across microbiome variables due to the repeated sampling design as well as potential confounding factors, but future work using both human interventional studies and animal experiments is needed to ascertain directionality in these associations. Additionally, while our measures of positive and negative emotions are derived from commonly used validated items, they have not been previously validated; therefore, our findings need to be replicated using validated measures of emotions. These limitations are balanced by considerable strengths. Notably, our study includes the collection of multiple stool samples per participant, detailed phenotyping of the participants, and a validated measure of emotion regulation.

In summary, we found evidence that positive and negative emotions, as well as emotion regulation strategies, are related to specific aspects of the gut microbiome for both phylogenetically diverse organisms and specific metabolic pathways including pantothenate and CoA and adenosine. Together, these results connect emotional functioning and the human gut microbiome, highlighting the critical importance of incorporating the microbiome in our understanding of emotion-related factors and their association with physical health.

## Supporting information

Ke et al. supplementary material Ke et al. supplementary material

## Data Availability

The data that support the findings of our study are available from Brigham and Women's Hospital and Harvard T.H. Chan School of Public Health. Restrictions apply to the availability of these data, which were used under license for our study. Data are available (https://sites.google.com/channing.harvard.edu/cohortdocs/) with the permission of Brigham and Women's Hospital and Harvard T.H. Chan School of Public Health.

## Supplementary material

The supplementary material for this article can be found at https://doi.org/10.1017/S0033291723000612
