# Supplementary material for: Gut feelings: associations of emotions and emotion regulation with the gut microbiome in women
Source: Psychol Med. 2023 Mar 21;53(15):7151–60. doi: 10.1017/S0033291723000612 (PMC10511660; doi:10.1017/S0033291723000612)
Supplement: Ke et al. supplementary material [file S0033291723000612sup001.docx]

**Gut feelings: Associations of emotions and emotion regulation with the gut microbiome in women**

Shanlin Ke^1*^, Anne-Josee Guimond^2,3*^, Shelley S. Tworoger^4,5^, Tianyi Huang^1^, Andrew T. Chan^6^, Yang-Yu Liu^1,7,#^, Laura D. Kubzansky^2#^.

*^1^ Channing Division of Network Medicine, Department of Medicine, Brigham and Women’s Hospital and Harvard Medical School, Boston, MA, 02115, USA.*

*^2^ Department of Social and Behavioral Sciences, Harvard T.H. Chan School of Public Health, Boston, MA, 02115, USA.*

*^3^ Lee Kum Sheung Center for Health and Happiness, Harvard T. H. Chan School of Public Health, 677 Huntington Avenue, Boston, MA 02115, USA.*

*^4^ Department of Cancer Epidemiology, Moffitt Cancer Center and Research Institute, Tampa, FL 33612, USA.*

*^5^Department of Epidemiology, Harvard T.H. Chan School of Public Health, Boston, MA 02115, USA.*

*^6^Clinical and Translational Epidemiology Unit, Massachusetts General Hospital and Harvard Medical School, Boston, MA 02115, USA.*

*^7^Center for Artificial Intelligence and Modeling, The Carl R. Woese Institute for Genomic Biology, University of Illinois at Urbana-Champaign, Champaign, IL 61801, USA.*

* These authors contributed equally.

#To whom correspondence should be addressed: Y.-Y.L. ([yyl@channing.harvard.edu)](mailto:yyl@channing.harvard.edu)) and L. D. K. ([lkubzans@hsph.harvard.edu)](mailto:lkubzans@hsph.harvard.edu))

**Supplementary Information**

**Supplemental information on diet measure**

**Supplemental information on gut microbiome analysis**

**Supplementary Table 1. Spearman correlations among emotion-related factors**

**Supplementary Table 2. Associations of emotion-related factors with microbial features in Figure 4.**

**Supplementary Table 3. Associations of emotion-related factors with metabolic pathways in Figure 5.**

**Supplementary Table 4. Associations of negative emotion with metabolic pathways. Only the statistically significant associations with *q* value *≤*0.25 (Benjamini-Hochberg adjusted *P* value) are reported here.**

**Diet measure.**

Since the gut microbial composition and biosynthetic capacity are responsive to host diet and microbes in turn influence nutrients reaching the host through the metabolism of food(David et al., 2014), we computed AHEI (the Alternate Healthy Eating Index 2010; a score that measures adherence to a diet pattern based on foods and nutrients most predictive of chronic disease risk) dietary score derived from the semiquantitative food frequency questionnaire (FFQ) data collected in 2013. The AHEI-2010 includes 11 variables: six components for which higher intakes are better (i.e., vegetables, fruit, whole grains, nuts and legumes, long-chain fats, and polyunsaturated fatty acids), one component for which moderate intake is better (i.e., alcohol), and four components that must be limited or avoided (i.e., sugar sweetened drinks and fruit juice, red/processed meat, trans fats, and sodium) (Chiuve et al., 2012). All AHEI-2010 components were scored on a 0-to-10 scale, and then the total AHEI-2010 score was created by summing each component’s score. The AHEI-2010 score can range from 0 (non-adherence) to 110 (perfect adherence), with higher scores representing healthier diet patterns (Varraso et al., 2015).

**Gut microbiome analysis**

In our study, each participant provided up to four fecal samples—one pair collected 24–72h apart and a second pair ~6 months later. Based on the requirements and usages of different analyses, we leveraged our microbiome data differently. Specifically, when considering alpha diversity, if participants provided multiple microbiome samples, we computed the average alpha diversity across samples for each participant. As mentioned in the Methods section, we assessed if the microbiome composition changed meaningfully over time using a permutational multivariate analysis of variance (PERMANOVA, a geometric partitioning of variation across a multivariate data cloud, defined explicitly in the space of a chosen dissimilarity measure, in response to one or more factors in an analysis of variance design) performed with the “adonis” function in R’s vegan package. The results revealed no significant differences over time with respect to both species (Fig.2c, *p*=0.999) and metabolic pathways (Fig2.d, p=0.877). As a result, we did not initiate analyses seeking to assess predictors of change in microbiome species or pathways over time. When identifying the gut microbiome associated host factors, we used cross-sectional data together with PERMANOVA to identify the host factors that are consistently associated with the overall structure of the gut microbiome over time. However, we did leverage the multiple measures of microbiome in other analyses as relevant. For example, to explore the potential links of the gut microbial species and pathways with emotions and emotion regulation strategies, we performed per-feature testing using MaAsLin2 (Microbiome Multivariable Associations with Linear Models) with the longitudinal microbiome data from all four time points. Specifically, all models included each participant’s identifier as a random effect to account for within-individual association from the study’s repeated sampling design, as well as occasional missing observation of microbiome samples at some time points.

**Supplementary Table 1.** Spearman correlations among emotion-related factors

|  | Positive emotions | Negative emotions | Cognitive reappraisal | Suppression |
| --- | --- | --- | --- | --- |
| Positive emotions | - |  |  |  |
| Negative emotions | -.49*** | - |  |  |
| Cognitive reappraisal | .24*** | -.14* | - |  |
| Suppression | -.30*** | .28*** | -.08 | - |

*Note.* **p*<.05 ***p* <.001 ****p* <.0001

**Supplementary Table 2.** Associations of top 10 species from each emotion-related factor showed in Figure 4.

| **Species** | **Emotional factors** | **Coefficient** | **Standard error** | ***p*-value** | ***q*-value** | **-Log10 (*q*-value)** | **Valence** |
| --- | --- | --- | --- | --- | --- | --- | --- |
| *Bacteroides faecis* | pos emo | -0.1632 | 0.0689 | 0.0188 | 0.2773 | 0.5571 | - |
| *Bacteroides xylanisolvens* | pos emo | 0.2197 | 0.0973 | 0.025 | 0.2949 | 0.5303 | + |
| *Bacteroides intestinalis* | pos emo | -0.1983 | 0.1033 | 0.0564 | 0.4306 | 0.3659 | - |
| *Bacteroides plebeius* | pos emo | -0.1258 | 0.0664 | 0.0596 | 0.4306 | 0.3659 | - |
| *Alistipes onderdonkii* | pos emo | 0.1109 | 0.0576 | 0.0557 | 0.4306 | 0.3659 | + |
| *Parabacteroides distasonis* | pos emo | 0.115 | 0.1169 | 0.3265 | 0.7754 | 0.1105 | + |
| *Ruminococcaceae bacterium D16* | pos emo | -0.2296 | 0.0615 | 0.0002 | 0.0740 | 1.1307 | - |
| *Firmicutes bacterium CAG 94* | pos emo | -0.1551 | 0.0616 | 0.0126 | 0.2382 | 0.6231 | - |
| *Lawsonibacter asaccharolyticus* | pos emo | -0.0980 | 0.0644 | 0.1296 | 0.5904 | 0.2289 | - |
| *Streptococcus parasanguinis* | pos emo | -0.0843 | 0.0652 | 0.1976 | 0.6898 | 0.1613 | - |
| *Firmicutes bacterium CAG 95* | pos emo | 0.0714 | 0.0561 | 0.2049 | 0.7037 | 0.1526 | + |
| *Clostridium bolteae CAG 59* | pos emo | -0.0342 | 0.0306 | 0.2659 | 0.7591 | 0.1197 | - |
| *Clostridium leptum* | pos emo | -0.0707 | 0.0657 | 0.2832 | 0.7706 | 0.1132 | - |
| *Anaeromassilibacillus sp An250* | pos emo | -0.0665 | 0.0645 | 0.3045 | 0.7706 | 0.1132 | - |
| *Firmicutes bacterium CAG 110* | pos emo | 0.1096 | 0.1015 | 0.2814 | 0.7706 | 0.1132 | + |
| *Clostridium scindens* | pos emo | 0.0314 | 0.0328 | 0.3398 | 0.7754 | 0.1105 | + |
| *Blautia hydrogenotrophica* | pos emo | -0.0519 | 0.0624 | 0.4069 | 0.8079 | 0.0926 | - |
| *Clostridium citroniae* | pos emo | 0.0451 | 0.0590 | 0.4455 | 0.8098 | 0.0916 | + |
| *Eisenbergiella massiliensis* | pos emo | 0.0513 | 0.0811 | 0.528 | 0.8283 | 0.0818 | + |
| *Sellimonas intestinalis* | pos emo | -0.0321 | 0.0587 | 0.5855 | 0.8507 | 0.0702 | - |
| *Holdemanella biformis* | pos emo | 0.0094 | 0.0507 | 0.8529 | 0.954 | 0.0204 | + |
| *Eubacterium siraeum* | pos emo | -0.017 | 0.1151 | 0.8826 | 0.9606 | 0.0175 | - |
| *Roseburia intestinalis* | pos emo | -0.0037 | 0.0994 | 0.9707 | 0.9925 | 0.0033 | - |
| *Coprococcus eutactus* | pos emo | -0.0032 | 0.0760 | 0.9670 | 0.9925 | 0.0033 | - |
| *Proteobacteria bacterium CAG 139* | pos emo | 0.2112 | 0.0889 | 0.0184 | 0.2773 | 0.5571 | + |
| *Bilophila wadsworthia* | pos emo | 0.1731 | 0.0748 | 0.0218 | 0.2931 | 0.533 | + |
| *Parasutterella excrementihominis* | pos emo | 0.1160 | 0.0882 | 0.1898 | 0.6663 | 0.1763 | + |
| *Akkermansia muciniphila* | pos emo | 0.2511 | 0.1144 | 0.0294 | 0.3152 | 0.5015 | + |
| *Bacteroides faecis* | neg emo | 0.1153 | 0.0670 | 0.0866 | 0.4637 | 0.3338 | + |
| *Bacteroides xylanisolvens* | neg emo | -0.1269 | 0.0944 | 0.1802 | 0.6077 | 0.2163 | - |
| *Bacteroides intestinalis* | neg emo | 0.1051 | 0.1000 | 0.2945 | 0.7158 | 0.1452 | + |
| *Bacteroides plebeius* | neg emo | 0.0341 | 0.0641 | 0.5946 | 0.8270 | 0.0825 | + |
| *Alistipes onderdonkii* | neg emo | -0.0187 | 0.0561 | 0.7392 | 0.8860 | 0.0526 | - |
| *Parabacteroides distasonis* | neg emo | -0.0333 | 0.1121 | 0.7669 | 0.9033 | 0.0442 | - |
| *Eubacterium siraeum* | neg emo | -0.3530 | 0.1094 | 0.0015 | 0.1275 | 0.8944 | - |
| *Clostridium bolteae CAG 59* | neg emo | 0.0968 | 0.0316 | 0.0025 | 0.1275 | 0.8944 | + |
| *Sellimonas intestinalis* | neg emo | 0.1765 | 0.0573 | 0.0023 | 0.1275 | 0.8944 | + |
| *Clostridium citroniae* | neg emo | 0.1680 | 0.0566 | 0.0033 | 0.1331 | 0.8758 | + |
| *Firmicutes bacterium CAG 94* | neg emo | 0.1764 | 0.0594 | 0.0033 | 0.1331 | 0.8758 | + |
| *Firmicutes bacterium CAG 95* | neg emo | -0.1477 | 0.0542 | 0.007 | 0.1604 | 0.7949 | - |
| *Ruminococcaceae bacterium D16* | neg emo | 0.156 | 0.0607 | 0.0108 | 0.1968 | 0.7061 | + |
| *Anaeromassilibacillus sp An250* | neg emo | 0.1567 | 0.0617 | 0.0118 | 0.2086 | 0.6806 | + |
| *Firmicutes bacterium CAG 110* | neg emo | -0.2421 | 0.0968 | 0.0132 | 0.2267 | 0.6445 | - |
| *Blautia hydrogenotrophica* | neg emo | 0.1443 | 0.0597 | 0.0165 | 0.2328 | 0.6330 | + |
| *Clostridium leptum* | neg emo | 0.1234 | 0.0644 | 0.0569 | 0.3893 | 0.4098 | + |
| *Streptococcus parasanguinis* | neg emo | 0.1112 | 0.0628 | 0.0781 | 0.4476 | 0.3491 | + |
| *Roseburia intestinalis* | neg emo | 0.1279 | 0.0953 | 0.1813 | 0.6077 | 0.2163 | + |
| *Coprococcus eutactus* | neg emo | -0.0921 | 0.0726 | 0.2059 | 0.612 | 0.2133 | - |
| *Clostridium scindens* | neg emo | 0.0389 | 0.0316 | 0.2190 | 0.6361 | 0.1965 | + |
| *Lawsonibacter asaccharolyticus* | neg emo | 0.0700 | 0.0620 | 0.2608 | 0.6882 | 0.1623 | + |
| *Holdemanella biformis* | neg emo | -0.0447 | 0.0484 | 0.3572 | 0.7462 | 0.1271 | - |
| *Eisenbergiella massiliensis* | neg emo | -0.0106 | 0.0788 | 0.8935 | 0.9737 | 0.0116 | - |
| *Bilophila wadsworthia* | neg emo | -0.1587 | 0.0721 | 0.0289 | 0.3012 | 0.5212 | - |
| *Proteobacteria bacterium CAG 139* | neg emo | -0.030 | 0.0873 | 0.7314 | 0.886 | 0.0526 | - |
| *Parasutterella excrementihominis* | neg emo | -0.0052 | 0.0857 | 0.9518 | 0.9907 | 0.004 | - |
| *Akkermansia muciniphila* | neg emo | -0.1450 | 0.1117 | 0.1958 | 0.6094 | 0.2151 | - |
| *Parabacteroides distasonis* | cog reappr | 0.2524 | 0.1126 | 0.026 | 0.3292 | 0.4825 | + |
| *Bacteroides faecis* | cog reappr | -0.0921 | 0.0684 | 0.1796 | 0.6342 | 0.1978 | - |
| *Bacteroides intestinalis* | cog reappr | -0.0828 | 0.1020 | 0.4182 | 0.8028 | 0.0954 | - |
| *Alistipes onderdonkii* | cog reappr | -0.0284 | 0.0571 | 0.6198 | 0.8984 | 0.0465 | - |
| *Bacteroides xylanisolvens* | cog reappr | 0.0459 | 0.0962 | 0.6339 | 0.9118 | 0.0401 | + |
| *Bacteroides plebeius* | cog reappr | 0.0052 | 0.0653 | 0.9372 | 0.9826 | 0.0076 | + |
| *Clostridium leptum* | cog reappr | -0.1570 | 0.0651 | 0.0167 | 0.2785 | 0.5552 | - |
| *Streptococcus parasanguinis* | cog reappr | -0.1269 | 0.0637 | 0.0478 | 0.4377 | 0.3588 | - |
| *Lawsonibacter asaccharolyticus* | cog reappr | -0.1170 | 0.0629 | 0.0641 | 0.4676 | 0.3301 | - |
| *Roseburia intestinalis* | cog reappr | -0.1759 | 0.0965 | 0.0698 | 0.4720 | 0.3260 | - |
| *Clostridium bolteae CAG 59* | cog reappr | -0.0589 | 0.0324 | 0.0709 | 0.4720 | 0.3260 | - |
| *Coprococcus eutactus* | cog reappr | 0.1320 | 0.0736 | 0.0743 | 0.4749 | 0.3234 | + |
| *Anaeromassilibacillus sp An250* | cog reappr | -0.1074 | 0.063 | 0.0900 | 0.4976 | 0.3031 | - |
| *Blautia hydrogenotrophica* | cog reappr | -0.0998 | 0.0611 | 0.1038 | 0.5163 | 0.2871 | - |
| *Firmicutes bacterium CAG 95* | cog reappr | 0.0797 | 0.0558 | 0.1547 | 0.5989 | 0.2227 | + |
| *Firmicutes bacterium CAG 110* | cog reappr | 0.1198 | 0.0996 | 0.2304 | 0.6980 | 0.1562 | + |
| *Eubacterium siraeum* | cog reappr | 0.1370 | 0.1139 | 0.2302 | 0.6980 | 0.1562 | + |
| *Sellimonas intestinalis* | cog reappr | -0.0611 | 0.0594 | 0.3050 | 0.7472 | 0.1266 | - |
| *Ruminococcaceae bacterium D16* | cog reappr | -0.0437 | 0.0627 | 0.4863 | 0.8295 | 0.0812 | - |
| *Eisenbergiella massiliensis* | cog reappr | -0.0485 | 0.0796 | 0.5434 | 0.8544 | 0.0683 | - |
| *Firmicutes bacterium CAG 94* | cog reappr | -0.0376 | 0.0617 | 0.5427 | 0.8544 | 0.0683 | - |
| *Clostridium scindens* | cog reappr | -0.0114 | 0.0322 | 0.7231 | 0.9268 | 0.033 | - |
| *Clostridium citroniae* | cog reappr | -0.0168 | 0.0585 | 0.7741 | 0.9377 | 0.0279 | - |
| *Holdemanella biformis* | cog reappr | -0.0082 | 0.0494 | 0.8691 | 0.967 | 0.0146 | - |
| *Parasutterella excrementihominis* | cog reappr | 0.1622 | 0.0865 | 0.0623 | 0.4658 | 0.3318 | + |
| *Proteobacteria bacterium CAG 139* | cog reappr | 0.1591 | 0.0881 | 0.0724 | 0.472 | 0.3260 | + |
| *Bilophila wadsworthia* | cog reappr | -0.0608 | 0.0740 | 0.4123 | 0.8028 | 0.0954 | - |
| *Akkermansia muciniphila* | cog reappr | -0.0387 | 0.1138 | 0.7343 | 0.9268 | 0.033 | - |
| *Alistipes onderdonkii* | suppr | -0.1292 | 0.0566 | 0.0235 | 0.3425 | 0.4654 | - |
| *Parabacteroides distasonis* | suppr | -0.2280 | 0.1132 | 0.0454 | 0.4199 | 0.3769 | - |
| *Bacteroides plebeius* | suppr | 0.1078 | 0.0651 | 0.0994 | 0.5609 | 0.2512 | + |
| *Bacteroides intestinalis* | suppr | -0.0914 | 0.1023 | 0.3731 | 0.8392 | 0.0761 | - |
| *Bacteroides faecis* | suppr | 0.0228 | 0.0689 | 0.7407 | 0.9288 | 0.0321 | + |
| *Bacteroides xylanisolvens* | suppr | -0.0121 | 0.0966 | 0.9005 | 0.9735 | 0.0117 | - |
| *Firmicutes bacterium CAG 95* | suppr | -0.1349 | 0.0555 | 0.0159 | 0.2621 | 0.5815 | - |
| *Eubacterium siraeum* | suppr | 0.2467 | 0.1132 | 0.0306 | 0.3579 | 0.4463 | + |
| *Ruminococcaceae bacterium D16* | suppr | 0.1184 | 0.0624 | 0.0592 | 0.4381 | 0.3584 | + |
| *Coprococcus eutactus* | suppr | -0.1360 | 0.0738 | 0.0668 | 0.4751 | 0.3232 | - |
| *Eisenbergiella massiliensis* | suppr | -0.1416 | 0.0793 | 0.0757 | 0.5034 | 0.2981 | - |
| *Clostridium scindens* | suppr | -0.0528 | 0.0321 | 0.1019 | 0.5651 | 0.2478 | - |
| *Holdemanella biformis* | suppr | 0.0761 | 0.0493 | 0.1245 | 0.6051 | 0.2182 | + |
| *Lawsonibacter asaccharolyticus* | suppr | -0.0757 | 0.0633 | 0.2331 | 0.7392 | 0.1312 | - |
| *Blautia hydrogenotrophica* | suppr | 0.0650 | 0.0615 | 0.2913 | 0.7821 | 0.1067 | + |
| *Sellimonas intestinalis* | suppr | 0.0622 | 0.0596 | 0.2981 | 0.7821 | 0.1067 | + |
| *Firmicutes bacterium CAG 110* | suppr | 0.0976 | 0.1000 | 0.3303 | 0.8219 | 0.0852 | + |
| *Anaeromassilibacillus sp An250* | suppr | 0.0592 | 0.0635 | 0.3528 | 0.8372 | 0.0772 | + |
| *Firmicutes bacterium CAG 94* | suppr | 0.0408 | 0.0618 | 0.5103 | 0.868 | 0.0615 | + |
| *Roseburia intestinalis* | suppr | -0.0516 | 0.0975 | 0.5974 | 0.8968 | 0.0473 | - |
| *Clostridium leptum* | suppr | 0.0310 | 0.0661 | 0.6392 | 0.918 | 0.0372 | + |
| *Clostridium citroniae* | suppr | 0.0239 | 0.0587 | 0.6844 | 0.9228 | 0.0349 | + |
| *Streptococcus parasanguinis* | suppr | 0.0256 | 0.0645 | 0.6912 | 0.9228 | 0.0349 | + |
| *Clostridium bolteae CAG 59* | suppr | 0.0053 | 0.0327 | 0.8724 | 0.9653 | 0.0153 | + |
| *Bilophila wadsworthia* | suppr | 0.0401 | 0.0743 | 0.5902 | 0.8903 | 0.0505 | + |
| *Proteobacteria bacterium CAG 139* | suppr | 0.0248 | 0.0891 | 0.7808 | 0.9381 | 0.0278 | + |
| *Parasutterella excrementihominis* | suppr | 0.0020 | 0.0875 | 0.9814 | 0.9941 | 0.0026 | + |
| *Akkermansia muciniphila* | suppr | 0.1431 | 0.1138 | 0.2100 | 0.7074 | 0.1504 | + |

Note: All the analyses in this table were conducted based on all 787 metagenomes collected from 206 participants. The results were calculated by applying linear mixed models in MaAsLin2 that included participant’s identifier as random effects and simultaneously adjusted for physical activity, BMI, and type 2 diabetes history. Only the *q* value *≤*0.25 (Benjamini-Hochberg adjusted *P* value) are considered as statistically significant associations. Positive emotions: pos emo; Negative emotions: neg emo; Cognitive reappraisal: cog reappr; Suppression: suppr; Positive: “+”; Negative: “-”.

**Supplementary Table 3.** Associations of top 10 metabolic pathways from each emotion-related factor showed in Figure 5.

| **Pathway** | **Emotional factors** | **Coefficient** | **Standard error** | ***p*-value** | ***q*-value** | **-Log10 (*q*-value)** | **Valence** |
| --- | --- | --- | --- | --- | --- | --- | --- |
| PWY0-1241: ADP-L-glycero-&beta;-D-manno-heptose biosynthesis | pos emo | 0.0671 | 0.0221 | 0.0028 | 0.0491 | 1.3085 | + |
| PWY0-1298: superpathway of pyrimidine deoxyribonucleosides degradation | pos emo | 0.0656 | 0.0282 | 0.0211 | 0.2075 | 0.683 | + |
| FUCCAT-PWY: fucose degradation | pos emo | 0.0475 | 0.0205 | 0.0219 | 0.2097 | 0.6783 | + |
| PWY-2941: L-lysine biosynthesis II | pos emo | 0.037 | 0.0178 | 0.0389 | 0.2854 | 0.5446 | + |
| RUMP-PWY: formaldehyde oxidation I | pos emo | 0.0688 | 0.0358 | 0.0564 | 0.3736 | 0.4276 | + |
| PWY-7046: 4-coumarate degradation (anaerobic) | pos emo | -0.0684 | 0.0361 | 0.0592 | 0.3864 | 0.4130 | - |
| FUC-RHAMCAT-PWY: superpathway of fucose and rhamnose degradation | pos emo | 0.0514 | 0.0273 | 0.0609 | 0.3894 | 0.4096 | + |
| PWY-6263: superpathway of menaquinol-8 biosynthesis II | pos emo | 0.0844 | 0.0452 | 0.063 | 0.3925 | 0.4061 | + |
| ALLANTOINDEG-PWY: superpathway of allantoin degradation in yeast | pos emo | -0.0468 | 0.0251 | 0.0638 | 0.3954 | 0.4029 | - |
| PWY66-398: TCA cycle III (animals) | pos emo | 0.0537 | 0.0299 | 0.0745 | 0.4134 | 0.3836 | + |
| PWY-7187: pyrimidine deoxyribonucleotides de novo biosynthesis II | pos emo | 0.0099 | 0.0061 | 0.1069 | 0.4796 | 0.3191 | + |
| PWY66-367: ketogenesis | pos emo | -0.0314 | 0.0196 | 0.1107 | 0.4862 | 0.3132 | - |
| PWY0-166: superpathway of pyrimidine deoxyribonucleotides de novo biosynthesis (E. coli) | pos emo | 0.0098 | 0.0062 | 0.1145 | 0.4890 | 0.3107 | + |
| P108-PWY: pyruvate fermentation to propanoate I | pos emo | 0.0354 | 0.0267 | 0.1865 | 0.6064 | 0.2172 | + |
| PWY-5863: superpathway of phylloquinol biosynthesis | pos emo | -0.0580 | 0.0444 | 0.1928 | 0.6081 | 0.2160 | - |
| PWY-5897: superpathway of menaquinol-11 biosynthesis | pos emo | -0.0568 | 0.0435 | 0.1926 | 0.6081 | 0.2160 | - |
| PWY-5898: superpathway of menaquinol-12 biosynthesis | pos emo | -0.0568 | 0.0435 | 0.1926 | 0.6081 | 0.2160 | - |
| PWY-5899: superpathway of menaquinol-13 biosynthesis | pos emo | -0.0568 | 0.0435 | 0.1926 | 0.6081 | 0.2160 | - |
| PWY-5791: 1,4-dihydroxy-2-naphthoate biosynthesis II (plants) | pos emo | -0.0584 | 0.0453 | 0.1986 | 0.6160 | 0.2104 | - |
| PWY-5837: 1,4-dihydroxy-2-naphthoate biosynthesis I | pos emo | -0.0584 | 0.0453 | 0.1986 | 0.6160 | 0.2104 | - |
| PWY-5861: superpathway of demethylmenaquinol-8 biosynthesis | pos emo | -0.0565 | 0.0439 | 0.1994 | 0.6160 | 0.2104 | - |
| PWY-5838: superpathway of menaquinol-8 biosynthesis I | pos emo | -0.0554 | 0.0430 | 0.1997 | 0.6160 | 0.2104 | - |
| PWY-3841: folate transformations II | pos emo | 0.0040 | 0.0043 | 0.3567 | 0.7563 | 0.1213 | + |
| PWY-7198: pyrimidine deoxyribonucleotides de novo biosynthesis IV | pos emo | -0.0180 | 0.0221 | 0.4152 | 0.7991 | 0.0974 | - |
| PEPTIDOGLYCANSYN-PWY: peptidoglycan biosynthesis I (meso-diaminopimelate containing) | pos emo | 0.0029 | 0.0038 | 0.4544 | 0.8266 | 0.0827 | + |
| PWY-6387: UDP-N-acetylmuramoyl-pentapeptide biosynthesis I (meso-diaminopimelate containing) | pos emo | 0.0027 | 0.0038 | 0.4778 | 0.8360 | 0.0778 | + |
| PWY-7371: 1,4-dihydroxy-6-naphthoate biosynthesis II | pos emo | 0.0450 | 0.0627 | 0.4741 | 0.8360 | 0.0778 | + |
| PWY-6385: peptidoglycan biosynthesis III (mycobacteria) | pos emo | 0.0024 | 0.0036 | 0.5045 | 0.8380 | 0.0767 | + |
| PWY-7315: dTDP-N-acetylthomosamine biosynthesis | pos emo | -0.0241 | 0.0497 | 0.6287 | 0.8804 | 0.0553 | - |
| PWY-5659: GDP-mannose biosynthesis | pos emo | -0.0029 | 0.0068 | 0.6665 | 0.9000 | 0.0458 | - |
| PWY-6386: UDP-N-acetylmuramoyl-pentapeptide biosynthesis II (lysine-containing) | pos emo | 0.0014 | 0.0037 | 0.7087 | 0.9044 | 0.0436 | + |
| PWY-6545: pyrimidine deoxyribonucleotides de novo biosynthesis III | pos emo | 0.0029 | 0.0079 | 0.7163 | 0.9056 | 0.0431 | + |
| PWY-6163: chorismate biosynthesis from 3-dehydroquinate | pos emo | 0.0009 | 0.0035 | 0.7924 | 0.9345 | 0.0294 | + |
| PWY-7357: thiamin formation from pyrithiamine and oxythiamine (yeast) | pos emo | -0.0015 | 0.0059 | 0.8046 | 0.9378 | 0.0279 | - |
| ARO-PWY: chorismate biosynthesis I | pos emo | 0.0008 | 0.0033 | 0.8082 | 0.9378 | 0.0279 | + |
| PWY-6609: adenine and adenosine salvage III | pos emo | -0.0008 | 0.0045 | 0.8668 | 0.9558 | 0.0196 | - |
| PWY-6897: thiamin salvage II | pos emo | 0.0008 | 0.0045 | 0.8651 | 0.9558 | 0.0196 | + |
| PWY-5136: fatty acid &beta;-oxidation II (peroxisome) | pos emo | 0.0054 | 0.0333 | 0.8709 | 0.9558 | 0.0196 | + |
| FAO-PWY: fatty acid &beta;-oxidation I | pos emo | 0.0034 | 0.0323 | 0.9152 | 0.9718 | 0.0124 | + |
| PWY-5686: UMP biosynthesis | pos emo | 0.0003 | 0.0041 | 0.9385 | 0.9803 | 0.0086 | + |
| PWY66-367: ketogenesis | neg emo | 0.0682 | 0.0187 | 0.0003 | 0.0134 | 1.8725 | + |
| PWY-6163: chorismate biosynthesis from 3-dehydroquinate | neg emo | -0.0106 | 0.0033 | 0.0013 | 0.0279 | 1.5546 | - |
| PEPTIDOGLYCANSYN-PWY: peptidoglycan biosynthesis I (meso-diaminopimelate containing) | neg emo | -0.0117 | 0.0036 | 0.0014 | 0.0289 | 1.5387 | - |
| ARO-PWY: chorismate biosynthesis I | neg emo | -0.0101 | 0.0031 | 0.0015 | 0.0294 | 1.5317 | - |
| PWY-6387: UDP-N-acetylmuramoyl-pentapeptide biosynthesis I (meso-diaminopimelate containing) | neg emo | -0.0113 | 0.0036 | 0.0016 | 0.0307 | 1.5123 | - |
| PWY-6386: UDP-N-acetylmuramoyl-pentapeptide biosynthesis II (lysine-containing) | neg emo | -0.0112 | 0.0035 | 0.0016 | 0.0307 | 1.5123 | - |
| PWY-5686: UMP biosynthesis | neg emo | -0.0122 | 0.0038 | 0.0017 | 0.0314 | 1.5037 | - |
| PWY-6609: adenine and adenosine salvage III | neg emo | -0.0134 | 0.0043 | 0.0018 | 0.0322 | 1.4924 | - |
| PWY-3841: folate transformations II | neg emo | -0.0127 | 0.0040 | 0.0019 | 0.0324 | 1.4899 | - |
| PWY-6385: peptidoglycan biosynthesis III (mycobacteria) | neg emo | -0.0102 | 0.0034 | 0.0027 | 0.0394 | 1.404 | - |
| ALLANTOINDEG-PWY: superpathway of allantoin degradation in yeast | neg emo | 0.0665 | 0.024 | 0.0061 | 0.0705 | 1.1519 | + |
| PWY-7371: 1,4-dihydroxy-6-naphthoate biosynthesis II | neg emo | -0.1400 | 0.0597 | 0.02 | 0.1509 | 0.8212 | - |
| PWY-6263: superpathway of menaquinol-8 biosynthesis II | neg emo | -0.0937 | 0.0436 | 0.0326 | 0.2007 | 0.6975 | - |
| PWY-7046: 4-coumarate degradation (anaerobic) | neg emo | 0.0730 | 0.0353 | 0.0402 | 0.2271 | 0.6438 | + |
| FAO-PWY: fatty acid &beta;-oxidation I | neg emo | 0.0592 | 0.0315 | 0.0617 | 0.2926 | 0.5338 | + |
| PWY-5136: fatty acid &beta;-oxidation II (peroxisome) | neg emo | 0.0595 | 0.0325 | 0.0683 | 0.3059 | 0.5145 | + |
| PWY-7198: pyrimidine deoxyribonucleotides de novo biosynthesis IV | neg emo | 0.0356 | 0.0213 | 0.096 | 0.3524 | 0.4529 | + |
| PWY-7187: pyrimidine deoxyribonucleotides de novo biosynthesis II | neg emo | -0.0087 | 0.0060 | 0.1491 | 0.4386 | 0.3579 | - |
| PWY-5791: 1,4-dihydroxy-2-naphthoate biosynthesis II (plants) | neg emo | 0.0550 | 0.0440 | 0.2126 | 0.5123 | 0.2905 | + |
| PWY-5837: 1,4-dihydroxy-2-naphthoate biosynthesis I | neg emo | 0.0550 | 0.0440 | 0.2126 | 0.5123 | 0.2905 | + |
| PWY-7315: dTDP-N-acetylthomosamine biosynthesis | neg emo | 0.0577 | 0.0477 | 0.2282 | 0.5329 | 0.2734 | + |
| PWY-5863: superpathway of phylloquinol biosynthesis | neg emo | 0.0520 | 0.0432 | 0.2296 | 0.5331 | 0.2732 | + |
| PWY-5861: superpathway of demethylmenaquinol-8 biosynthesis | neg emo | 0.0498 | 0.0426 | 0.2441 | 0.5465 | 0.2624 | + |
| PWY-5897: superpathway of menaquinol-11 biosynthesis | neg emo | 0.0489 | 0.0423 | 0.249 | 0.5494 | 0.2601 | + |
| PWY-5898: superpathway of menaquinol-12 biosynthesis | neg emo | 0.0489 | 0.0423 | 0.249 | 0.5494 | 0.2601 | + |
| PWY-5899: superpathway of menaquinol-13 biosynthesis | neg emo | 0.0489 | 0.0423 | 0.249 | 0.5494 | 0.2601 | + |
| PWY-5838: superpathway of menaquinol-8 biosynthesis I | neg emo | 0.0467 | 0.0419 | 0.2659 | 0.5772 | 0.2387 | + |
| PWY-6897: thiamin salvage II | neg emo | -0.0048 | 0.0044 | 0.2733 | 0.5855 | 0.2324 | - |
| PWY0-166: superpathway of pyrimidine deoxyribonucleotides de novo biosynthesis (E. coli) | neg emo | -0.0061 | 0.0061 | 0.3148 | 0.6211 | 0.2068 | - |
| RUMP-PWY: formaldehyde oxidation I | neg emo | -0.0281 | 0.0351 | 0.4249 | 0.7087 | 0.1496 | - |
| FUCCAT-PWY: fucose degradation | neg emo | -0.0156 | 0.0202 | 0.4389 | 0.7127 | 0.1471 | - |
| PWY-7357: thiamin formation from pyrithiamine and oxythiamine (yeast) | neg emo | -0.0042 | 0.0057 | 0.4586 | 0.7306 | 0.1363 | - |
| PWY-6545: pyrimidine deoxyribonucleotides de novo biosynthesis III | neg emo | -0.0053 | 0.0077 | 0.4891 | 0.7549 | 0.1221 | - |
| P108-PWY: pyruvate fermentation to propanoate I | neg emo | -0.0159 | 0.0261 | 0.5415 | 0.7872 | 0.1039 | - |
| FUC-RHAMCAT-PWY: superpathway of fucose and rhamnose degradation | neg emo | 0.0154 | 0.0266 | 0.5638 | 0.7872 | 0.1039 | + |
| PWY66-398: TCA cycle III (animals) | neg emo | -0.0146 | 0.0296 | 0.6220 | 0.8153 | 0.0887 | - |
| PWY0-1298: superpathway of pyrimidine deoxyribonucleosides degradation | neg emo | 0.0135 | 0.0281 | 0.6315 | 0.821 | 0.0856 | + |
| PWY-2941: L-lysine biosynthesis II | neg emo | -0.0068 | 0.0173 | 0.6965 | 0.8454 | 0.0729 | - |
| PWY0-1241: ADP-L-glycero-&beta;-D-manno-heptose biosynthesis | neg emo | -0.0019 | 0.022 | 0.9304 | 0.9657 | 0.0152 | - |
| PWY-5659: GDP-mannose biosynthesis | neg emo | -0.0002 | 0.0066 | 0.9708 | 0.9828 | 0.0075 | - |
| PWY-5838: superpathway of menaquinol-8 biosynthesis I | cog reappr | -0.0904 | 0.042 | 0.0326 | 0.2588 | 0.5871 | - |
| PWY-5861: superpathway of demethylmenaquinol-8 biosynthesis | cog reappr | -0.0912 | 0.0428 | 0.0344 | 0.2660 | 0.5752 | - |
| PWY-7198: pyrimidine deoxyribonucleotides de novo biosynthesis IV | cog reappr | -0.0454 | 0.0215 | 0.0359 | 0.2700 | 0.5687 | - |
| PWY-5897: superpathway of menaquinol-11 biosynthesis | cog reappr | -0.0880 | 0.0425 | 0.0394 | 0.2783 | 0.5555 | - |
| PWY-5898: superpathway of menaquinol-12 biosynthesis | cog reappr | -0.0880 | 0.0425 | 0.0394 | 0.2783 | 0.5555 | - |
| PWY-5899: superpathway of menaquinol-13 biosynthesis | cog reappr | -0.0880 | 0.0425 | 0.0394 | 0.2783 | 0.5555 | - |
| PWY-5863: superpathway of phylloquinol biosynthesis | cog reappr | -0.0885 | 0.0434 | 0.0426 | 0.2932 | 0.5328 | - |
| PWY-5791: 1,4-dihydroxy-2-naphthoate biosynthesis II (plants) | cog reappr | -0.0891 | 0.0442 | 0.0452 | 0.3048 | 0.5160 | - |
| PWY-5837: 1,4-dihydroxy-2-naphthoate biosynthesis I | cog reappr | -0.0891 | 0.0442 | 0.0452 | 0.3048 | 0.5160 | - |
| PWY-7371: 1,4-dihydroxy-6-naphthoate biosynthesis II | cog reappr | 0.1205 | 0.0607 | 0.0486 | 0.3218 | 0.4924 | + |
| ALLANTOINDEG-PWY: superpathway of allantoin degradation in yeast | cog reappr | -0.0385 | 0.0247 | 0.1204 | 0.4797 | 0.3190 | - |
| PWY-6263: superpathway of menaquinol-8 biosynthesis II | cog reappr | 0.0636 | 0.0445 | 0.1546 | 0.5421 | 0.2659 | + |
| PWY-5659: GDP-mannose biosynthesis | cog reappr | -0.0088 | 0.0066 | 0.1806 | 0.5897 | 0.2294 | - |
| PWY66-367: ketogenesis | cog reappr | -0.0194 | 0.0195 | 0.3203 | 0.7263 | 0.1389 | - |
| RUMP-PWY: formaldehyde oxidation I | cog reappr | 0.0272 | 0.0355 | 0.4438 | 0.8155 | 0.0886 | + |
| PWY-2941: L-lysine biosynthesis II | cog reappr | -0.0107 | 0.0175 | 0.5434 | 0.8505 | 0.0703 | - |
| PWY-7187: pyrimidine deoxyribonucleotides de novo biosynthesis II | cog reappr | -0.0035 | 0.0061 | 0.5669 | 0.8525 | 0.0693 | - |
| PWY-3841: folate transformations II | cog reappr | -0.0024 | 0.0042 | 0.577 | 0.8525 | 0.0693 | - |
| PWY66-398: TCA cycle III (animals) | cog reappr | 0.0167 | 0.0301 | 0.5799 | 0.8554 | 0.0679 | + |
| PWY-5136: fatty acid &beta;-oxidation II (peroxisome) | cog reappr | -0.0177 | 0.0332 | 0.5953 | 0.8601 | 0.0655 | - |
| PWY-7315: dTDP-N-acetylthomosamine biosynthesis | cog reappr | -0.0248 | 0.0486 | 0.6111 | 0.8605 | 0.0652 | - |
| PWY-6545: pyrimidine deoxyribonucleotides de novo biosynthesis III | cog reappr | -0.0040 | 0.0078 | 0.6086 | 0.8605 | 0.0652 | - |
| FAO-PWY: fatty acid &beta;-oxidation I | cog reappr | -0.0153 | 0.0322 | 0.6347 | 0.8721 | 0.0594 | - |
| PWY-6163: chorismate biosynthesis from 3-dehydroquinate | cog reappr | -0.0014 | 0.0034 | 0.6895 | 0.8966 | 0.0474 | - |
| P108-PWY: pyruvate fermentation to propanoate I | cog reappr | 0.0106 | 0.0259 | 0.6812 | 0.8966 | 0.0474 | + |
| PWY-7357: thiamin formation from pyrithiamine and oxythiamine (yeast) | cog reappr | 0.0019 | 0.0057 | 0.735 | 0.9155 | 0.0383 | + |
| ARO-PWY: chorismate biosynthesis I | cog reappr | -0.0009 | 0.0033 | 0.7887 | 0.9351 | 0.0291 | - |
| PWY0-166: superpathway of pyrimidine deoxyribonucleotides de novo biosynthesis (E. coli) | cog reappr | -0.0014 | 0.0062 | 0.8235 | 0.9536 | 0.0206 | - |
| PWY-6385: peptidoglycan biosynthesis III (mycobacteria) | cog reappr | 0.0007 | 0.0035 | 0.8456 | 0.9575 | 0.0189 | + |
| PWY-6386: UDP-N-acetylmuramoyl-pentapeptide biosynthesis II (lysine-containing) | cog reappr | 0.0007 | 0.0036 | 0.8504 | 0.9575 | 0.0189 | + |
| PEPTIDOGLYCANSYN-PWY: peptidoglycan biosynthesis I (meso-diaminopimelate containing) | cog reappr | 0.0007 | 0.0038 | 0.845 | 0.9575 | 0.0189 | + |
| PWY-5686: UMP biosynthesis | cog reappr | 0.0008 | 0.0040 | 0.8473 | 0.9575 | 0.0189 | + |
| PWY-6897: thiamin salvage II | cog reappr | 0.0008 | 0.0044 | 0.851 | 0.9575 | 0.0189 | + |
| FUCCAT-PWY: fucose degradation | cog reappr | -0.0040 | 0.0205 | 0.8449 | 0.9575 | 0.0189 | - |
| PWY0-1298: superpathway of pyrimidine deoxyribonucleosides degradation | cog reappr | 0.0056 | 0.0284 | 0.8449 | 0.9575 | 0.0189 | + |
| PWY0-1241: ADP-L-glycero-&beta;-D-manno-heptose biosynthesis | cog reappr | -0.0028 | 0.0223 | 0.9019 | 0.967 | 0.0146 | - |
| PWY-6387: UDP-N-acetylmuramoyl-pentapeptide biosynthesis I (meso-diaminopimelate containing) | cog reappr | 0.0003 | 0.0037 | 0.9333 | 0.9764 | 0.0104 | + |
| PWY-6609: adenine and adenosine salvage III | cog reappr | -0.0002 | 0.0044 | 0.9665 | 0.9872 | 0.0056 | - |
| FUC-RHAMCAT-PWY: superpathway of fucose and rhamnose degradation | cog reappr | 0.0011 | 0.0270 | 0.9661 | 0.9872 | 0.0056 | + |
| PWY-7046: 4-coumarate degradation (anaerobic) | cog reappr | -0.0009 | 0.0362 | 0.98 | 0.9898 | 0.0044 | - |
| PWY-7187: pyrimidine deoxyribonucleotides de novo biosynthesis II | suppr | -0.0157 | 0.006 | 0.0092 | 0.1098 | 0.9593 | - |
| P108-PWY: pyruvate fermentation to propanoate I | suppr | -0.0616 | 0.0256 | 0.0169 | 0.1669 | 0.7774 | - |
| PWY-7315: dTDP-N-acetylthomosamine biosynthesis | suppr | 0.1145 | 0.0481 | 0.0183 | 0.1786 | 0.748 | + |
| PWY-7357: thiamin formation from pyrithiamine and oxythiamine (yeast) | suppr | 0.0116 | 0.0057 | 0.0434 | 0.3048 | 0.516 | + |
| FAO-PWY: fatty acid &beta;-oxidation I | suppr | 0.0640 | 0.0320 | 0.0470 | 0.3212 | 0.4933 | + |
| PWY-5136: fatty acid &beta;-oxidation II (peroxisome) | suppr | 0.0650 | 0.0330 | 0.0500 | 0.3359 | 0.4738 | + |
| PWY-6897: thiamin salvage II | suppr | 0.0085 | 0.0044 | 0.0532 | 0.3473 | 0.4593 | + |
| PWY0-166: superpathway of pyrimidine deoxyribonucleotides de novo biosynthesis (E. coli) | suppr | -0.0115 | 0.0061 | 0.0617 | 0.3793 | 0.421 | - |
| PWY-6545: pyrimidine deoxyribonucleotides de novo biosynthesis III | suppr | -0.0142 | 0.0077 | 0.0684 | 0.3927 | 0.406 | - |
| PWY-5659: GDP-mannose biosynthesis | suppr | -0.0119 | 0.0065 | 0.0704 | 0.3949 | 0.4035 | - |
| PWY0-1241: ADP-L-glycero-&beta;-D-manno-heptose biosynthesis | suppr | -0.0333 | 0.0223 | 0.1359 | 0.534 | 0.2724 | - |
| RUMP-PWY: formaldehyde oxidation I | suppr | -0.0499 | 0.0354 | 0.1603 | 0.5687 | 0.2451 | - |
| PWY66-398: TCA cycle III (animals) | suppr | 0.0385 | 0.0300 | 0.2019 | 0.6381 | 0.1951 | + |
| PWY0-1298: superpathway of pyrimidine deoxyribonucleosides degradation | suppr | -0.0347 | 0.0284 | 0.2229 | 0.6676 | 0.1755 | - |
| ALLANTOINDEG-PWY: superpathway of allantoin degradation in yeast | suppr | 0.0266 | 0.0248 | 0.2838 | 0.7324 | 0.1353 | + |
| PWY-7046: 4-coumarate degradation (anaerobic) | suppr | 0.0385 | 0.0362 | 0.2884 | 0.7329 | 0.135 | + |
| ARO-PWY: chorismate biosynthesis I | suppr | -0.0033 | 0.0033 | 0.3097 | 0.7518 | 0.1239 | - |
| PWY-2941: L-lysine biosynthesis II | suppr | -0.0178 | 0.0175 | 0.3104 | 0.7523 | 0.1236 | - |
| PWY66-367: ketogenesis | suppr | 0.0196 | 0.0195 | 0.3157 | 0.7553 | 0.1219 | + |
| PWY-6263: superpathway of menaquinol-8 biosynthesis II | suppr | -0.0406 | 0.0448 | 0.3661 | 0.7816 | 0.1070 | - |
| PWY-5838: superpathway of menaquinol-8 biosynthesis I | suppr | 0.0390 | 0.0425 | 0.3597 | 0.7816 | 0.1070 | + |
| PWY-5861: superpathway of demethylmenaquinol-8 biosynthesis | suppr | 0.0395 | 0.0433 | 0.3622 | 0.7816 | 0.1070 | + |
| PWY-7371: 1,4-dihydroxy-6-naphthoate biosynthesis II | suppr | -0.0535 | 0.0614 | 0.3848 | 0.7909 | 0.1019 | - |
| PWY-5863: superpathway of phylloquinol biosynthesis | suppr | 0.0349 | 0.0439 | 0.4271 | 0.813 | 0.0899 | + |
| PWY-5897: superpathway of menaquinol-11 biosynthesis | suppr | 0.0338 | 0.0429 | 0.4324 | 0.8137 | 0.0895 | + |
| PWY-5898: superpathway of menaquinol-12 biosynthesis | suppr | 0.0338 | 0.0429 | 0.4324 | 0.8137 | 0.0895 | + |
| PWY-5899: superpathway of menaquinol-13 biosynthesis | suppr | 0.0338 | 0.0429 | 0.4324 | 0.8137 | 0.0895 | + |
| PWY-5791: 1,4-dihydroxy-2-naphthoate biosynthesis II (plants) | suppr | 0.0349 | 0.0447 | 0.4357 | 0.8137 | 0.0895 | + |
| PWY-5837: 1,4-dihydroxy-2-naphthoate biosynthesis I | suppr | 0.0349 | 0.0447 | 0.4357 | 0.8137 | 0.0895 | + |
| FUC-RHAMCAT-PWY: superpathway of fucose and rhamnose degradation | suppr | 0.0209 | 0.027 | 0.4395 | 0.8137 | 0.0895 | + |
| FUCCAT-PWY: fucose degradation | suppr | 0.0113 | 0.0205 | 0.5817 | 0.8556 | 0.0677 | + |
| PWY-6163: chorismate biosynthesis from 3-dehydroquinate | suppr | -0.0018 | 0.0034 | 0.5924 | 0.8622 | 0.0644 | - |
| PWY-7198: pyrimidine deoxyribonucleotides de novo biosynthesis IV | suppr | -0.0107 | 0.0218 | 0.6241 | 0.874 | 0.0585 | - |
| PWY-3841: folate transformations II | suppr | -0.0019 | 0.0042 | 0.6577 | 0.8851 | 0.053 | - |
| PWY-6387: UDP-N-acetylmuramoyl-pentapeptide biosynthesis I (meso-diaminopimelate containing) | suppr | -0.0009 | 0.0037 | 0.8112 | 0.9341 | 0.0296 | - |
| PEPTIDOGLYCANSYN-PWY: peptidoglycan biosynthesis I (meso-diaminopimelate containing) | suppr | -0.0006 | 0.0038 | 0.8671 | 0.9515 | 0.0216 | - |
| PWY-6609: adenine and adenosine salvage III | suppr | 0.0004 | 0.0044 | 0.9265 | 0.9712 | 0.0127 | + |
| PWY-6386: UDP-N-acetylmuramoyl-pentapeptide biosynthesis II (lysine-containing) | suppr | 0.0003 | 0.0036 | 0.9377 | 0.9749 | 0.0111 | + |
| PWY-6385: peptidoglycan biosynthesis III (mycobacteria) | suppr | -0.0002 | 0.0035 | 0.9545 | 0.9811 | 0.0083 | - |
| PWY-5686: UMP biosynthesis | suppr | 0.0002 | 0.004 | 0.9625 | 0.9824 | 0.0077 | + |

Note: All the analyses in this table were conducted based on all 787 metagenomes collected from 206 participants. The results were calculated by applying linear mixed models in MaAsLin2 that included participant’s identifier as random effects and simultaneously adjusted for physical activity, BMI, and type 2 diabetes history. Only the *q* value *≤*0.25 (Benjamini-Hochberg adjusted *P* value) are considered as statistically significant associations. Positive emotions: pos emo; Negative emotions: neg emo; Cognitive reappraisal: cog reappr; Suppression: suppr; Positive: “+”; Negative: “-”.

**Supplementary Table 4.** Associations of negative emotions with additional metabolic pathways beyond the top 10 showed in Figure 5. Only the statistically significant associations with *q* value *≤*0.25 (Benjamini-Hochberg adjusted *P* value) are reported here.

| **Pathway** | **Coefficient** | **Standard error** | ***p*-value** | ***q*-value** | **-Log10 (*q*-value)** | **Valence** |
| --- | --- | --- | --- | --- | --- | --- |
| PWY-7219: adenosine ribonucleotides de novo biosynthesis | -0.0118 | 0.0039 | 0.0028 | 0.0395 | 1.4035 | - |
| PWY-7229: superpathway of adenosine nucleotides de novo biosynthesis I | -0.0078 | 0.0026 | 0.0028 | 0.0399 | 1.3987 | - |
| PWY-7221: guanosine ribonucleotides de novo biosynthesis | -0.0101 | 0.0035 | 0.0042 | 0.056 | 1.2520 | - |
| COA-PWY-1: coenzyme A biosynthesis II (mammalian) | -0.0106 | 0.0037 | 0.005 | 0.0611 | 1.2142 | - |
| PWY-6891: thiazole biosynthesis II (Bacillus) | 0.0873 | 0.0309 | 0.0052 | 0.0633 | 1.1985 | + |
| 1CMET2-PWY: N10-formyl-tetrahydrofolate biosynthesis | -0.0096 | 0.0034 | 0.0058 | 0.0692 | 1.1599 | - |
| PWY3DJ-35471: L-ascorbate biosynthesis IV | 0.0643 | 0.0231 | 0.0059 | 0.0692 | 1.1599 | + |
| PWY-6126: superpathway of adenosine nucleotides de novo biosynthesis II | -0.0079 | 0.0029 | 0.0066 | 0.0738 | 1.1320 | - |
| PWY-2942: L-lysine biosynthesis III | -0.0091 | 0.0033 | 0.0067 | 0.0741 | 1.1303 | - |
| PWY-6123: inosine-5'-phosphate biosynthesis I | -0.0115 | 0.0043 | 0.0085 | 0.0864 | 1.0633 | - |
| P441-PWY: superpathway of N-acetylneuraminate degradation | 0.0542 | 0.0205 | 0.0087 | 0.0876 | 1.0574 | + |
| PWY-6700: queuosine biosynthesis | -0.011 | 0.0042 | 0.0093 | 0.0926 | 1.0335 | - |
| COMPLETE-ARO-PWY: superpathway of aromatic amino acid biosynthesis | -0.0073 | 0.0028 | 0.0105 | 0.1011 | 0.9951 | - |
| PWY-6124: inosine-5'-phosphate biosynthesis II | -0.012 | 0.0047 | 0.0107 | 0.1021 | 0.9909 | - |
| P221-PWY: octane oxidation | 0.1019 | 0.0396 | 0.0108 | 0.1023 | 0.9902 | + |
| PWY-7199: pyrimidine deoxyribonucleosides salvage | -0.0116 | 0.0045 | 0.0116 | 0.1081 | 0.9661 | - |
| P163-PWY: L-lysine fermentation to acetate and butanoate | 0.0914 | 0.0359 | 0.0116 | 0.1081 | 0.9661 | + |
| PWY-5667: CDP-diacylglycerol biosynthesis I | -0.0082 | 0.0033 | 0.0123 | 0.1111 | 0.9544 | - |
| PWY0-1319: CDP-diacylglycerol biosynthesis II | -0.0082 | 0.0033 | 0.0123 | 0.1111 | 0.9544 | - |
| PWY-6121: 5-aminoimidazole ribonucleotide biosynthesis I | -0.0076 | 0.0031 | 0.0143 | 0.1243 | 0.9056 | - |
| PWY-5097: L-lysine biosynthesis VI | -0.0081 | 0.0033 | 0.0147 | 0.1259 | 0.8999 | - |
| PWY-6895: superpathway of thiamin diphosphate biosynthesis II | 0.0632 | 0.0259 | 0.0156 | 0.1309 | 0.8830 | + |
| PWY-5913: TCA cycle VI (obligate autotrophs) | 0.0976 | 0.0406 | 0.017 | 0.1395 | 0.8554 | + |
| PWY-724: superpathway of L-lysine, L-threonine and L-methionine biosynthesis II | -0.0066 | 0.0028 | 0.0172 | 0.1401 | 0.8537 | - |
| GLYCOLYSIS-E-D: superpathway of glycolysis and Entner-Doudoroff | 0.0250 | 0.0105 | 0.0178 | 0.1423 | 0.8468 | + |
| PWY-7117: C4 photosynthetic carbon assimilation cycle, PEPCK type | 0.0956 | 0.0402 | 0.0185 | 0.1468 | 0.8333 | + |
| PWY-6690: cinnamate and 3-hydroxycinnamate degradation to 2-oxopent-4-enoate | 0.0766 | 0.0324 | 0.0190 | 0.1476 | 0.8309 | + |
| HCAMHPDEG-PWY: 3-phenylpropanoate and 3-(3-hydroxyphenyl)propanoate degradation to 2-oxopent-4-enoate | 0.0766 | 0.0324 | 0.0190 | 0.1476 | 0.8309 | + |
| PWY-7204: pyridoxal 5'-phosphate salvage II (plants) | 0.0869 | 0.0367 | 0.0188 | 0.1476 | 0.8309 | + |
| PWY-7332: superpathway of UDP-N-acetylglucosamine-derived O-antigen building blocks biosynthesis | 0.1165 | 0.0494 | 0.0192 | 0.1479 | 0.8301 | + |
| PWY-7115: C4 photosynthetic carbon assimilation cycle, NAD-ME type | 0.0988 | 0.0423 | 0.0206 | 0.1537 | 0.8133 | + |
| PWY-6901: superpathway of glucose and xylose degradation | 0.0188 | 0.0081 | 0.0211 | 0.1545 | 0.8109 | + |
| PWY-241: C4 photosynthetic carbon assimilation cycle, NADP-ME type | 0.0946 | 0.0408 | 0.0215 | 0.1545 | 0.8109 | + |
| PWY-5189: tetrapyrrole biosynthesis II (from glycine) | 0.0961 | 0.0415 | 0.0216 | 0.1545 | 0.8109 | + |
| PWY0-1277: 3-phenylpropanoate and 3-(3-hydroxyphenyl)propanoate degradation | 0.0663 | 0.0287 | 0.0218 | 0.1552 | 0.8092 | + |
| PWY0-1296: purine ribonucleosides degradation | -0.0171 | 0.0074 | 0.0227 | 0.1602 | 0.7953 | - |
| PWY-6628: superpathway of L-phenylalanine biosynthesis | 0.0962 | 0.0423 | 0.024 | 0.1645 | 0.7838 | + |
| PWY0-1338: polymyxin resistance | 0.0707 | 0.0315 | 0.0261 | 0.17 | 0.7695 | + |
| PANTOSYN-PWY: pantothenate and coenzyme A biosynthesis I | -0.0076 | 0.0035 | 0.0314 | 0.1939 | 0.7124 | - |
| COA-PWY: coenzyme A biosynthesis I | -0.0082 | 0.0038 | 0.0331 | 0.2027 | 0.6932 | - |
| PWY-6572: chondroitin sulfate degradation I (bacterial) | -0.0855 | 0.0402 | 0.0344 | 0.2061 | 0.6859 | - |
| PWY-6122: 5-aminoimidazole ribonucleotide biosynthesis II | -0.0068 | 0.0032 | 0.0346 | 0.2061 | 0.6859 | - |
| PWY-6277: superpathway of 5-aminoimidazole ribonucleotide biosynthesis | -0.0068 | 0.0032 | 0.0346 | 0.2061 | 0.6859 | - |
| BRANCHED-CHAIN-AA-SYN-PWY: superpathway of branched amino acid biosynthesis | -0.0073 | 0.0035 | 0.0357 | 0.2090 | 0.6798 | - |
| PWY4LZ-257: superpathway of fermentation (Chlamydomonas reinhardtii) | 0.0738 | 0.0035 | 0.0360 | 0.2090 | 0.6798 | + |
| KDO-NAGLIPASYN-PWY: superpathway of (Kdo)2-lipid A biosynthesis | 0.0627 | 0.0304 | 0.0403 | 0.2271 | 0.6438 | + |
| P161-PWY: acetylene degradation | 0.0743 | 0.0363 | 0.0417 | 0.2325 | 0.6335 | + |
| HISTSYN-PWY: L-histidine biosynthesis | -0.0082 | 0.0040 | 0.0429 | 0.2369 | 0.6255 | - |
| PWY-4242: pantothenate and coenzyme A biosynthesis III | -0.0084 | 0.0042 | 0.0449 | 0.2453 | 0.6102 | - |

Note: All the analyses in this table were conducted based on all 787 metagenomes collected from 206 participants. The results were calculated by applying linear mixed models in MaAsLin2 that included participant’s identifier as random effects and simultaneously adjusted for physical activity, BMI, and type 2 diabetes history. Positive emotions: pos emo; Negative emotions: neg emo; Cognitive reappraisal: cog reappr; Suppression: suppr; Positive: “+”; Negative: “-”.

**References**

Chiuve, S. E., Fung, T. T., Rimm, E. B., Hu, F. B., McCullough, M. L., Wang, M., . . . Willett, W. C. (2012). Alternative dietary indices both strongly predict risk of chronic disease. *The* *Journal of Nutrition, 142*(6), 1009-1018. doi:10.3945/jn.111.157222

David, L. A., Maurice, C. F., Carmody, R. N., Gootenberg, D. B., Button, J. E., Wolfe, B. E., . . . Turnbaugh, P. J. (2014). Diet rapidly and reproducibly alters the human gut microbiome. *Nature, 505*(7484), 559-563. doi:10.1038/nature12820

Varraso, R., Chiuve, S. E., Fung, T. T., Barr, R. G., Hu, F. B., Willett, W. C., & Camargo, C. A. (2015). Alternate Healthy Eating Index 2010 and risk of chronic obstructive pulmonary disease among US women and men: prospective study. *BMJ, 350*, h286. doi:10.1136/bmj.h286
